# Mechanical suppression of breast cancer cell invasion and paracrine signaling to osteoclasts requires nucleo-cytoskeletal connectivity

**DOI:** 10.1038/s41413-020-00111-3

**Published:** 2020-11-17

**Authors:** Xin Yi, Laura E. Wright, Gabriel M. Pagnotti, Gunes Uzer, Katherine M. Powell, Joseph M. Wallace, Uma Sankar, Clinton T. Rubin, Khalid Mohammad, Theresa A. Guise, William R. Thompson

**Affiliations:** 1 Department of Physical Therapy, School of Health and Rehabilitation Sciences, Indiana University, Indianapolis, IN 46202 USA; 2 Department of Medicine, Division of Endocrinology, School of Medicine, Indiana University, Indianapolis, IN 46202 USA; 3 Department of Mechanical and Biomedical Engineering, Boise State University, Boise, ID 83725 USA; 4 Department of Biomedical Engineering, Purdue School of Engineering and Technology, Purdue University, Indianapolis, IN 46202 USA; 5 Department of Anatomy & Cell Biology, Indiana University, Indianapolis, IN 46202 USA; 6 Department of Biomedical Engineering, Stony Brook University, Stony Brook, NY 11794 USA

**Keywords:** Bone cancer, Bone quality and biomechanics

## Abstract

Exercise benefits the musculoskeletal system and reduces the effects of cancer. The effects of exercise are multifactorial, where metabolic changes and tissue adaptation influence outcomes. Mechanical signals, a principal component of exercise, are anabolic to the musculoskeletal system and restrict cancer progression. We examined the mechanisms through which cancer cells sense and respond to low-magnitude mechanical signals introduced in the form of vibration. Low-magnitude, high-frequency vibration was applied to human breast cancer cells in the form of low-intensity vibration (LIV). LIV decreased matrix invasion and impaired secretion of osteolytic factors PTHLH, IL-11, and RANKL. Furthermore, paracrine signals from mechanically stimulated cancer cells, reduced osteoclast differentiation and resorptive capacity. Disconnecting the nucleus by knockdown of *SUN1* and *SUN2* impaired LIV-mediated suppression of invasion and osteolytic factor secretion. LIV increased cell stiffness; an effect dependent on the LINC complex. These data show that mechanical vibration reduces the metastatic potential of human breast cancer cells, where the nucleus serves as a mechanosensory apparatus to alter cell structure and intercellular signaling.

## Introduction

Physical activity has beneficial consequences on nearly every organ system. In addition to the positive effects of exercise on cardiovascular^1^ and musculoskeletal health,^2^ regular physical activity is associated with a reduced risk of colon, endometrial, and breast cancers.^3,4^ Women who exercise, at a moderate intensity for 3–4 h per week, have a 30%–40% reduced risk of breast cancer, compared to sedentary women.^5^ In addition, physical activity is associated with decreased cancer mortality^6^ and reduced tumor size in mice that ran long distances.^7^ Importantly, there are beneficial effects of physical activity on cancer outcomes are realized even at low doses.^8^ While these studies highlight the positive effects of exercise on cancer, the underlying mechanisms remain largely unknown.

Physical activity inherently involves repetitive bouts of physical movement, altering whole-body homeostasis with subsequent adaptations at the cell, tissue, and organ levels.^2^ The beneficial effects of physical activity on cancer seems, at least partially, due to metabolic and immune effects. Physical activity results in reduced insulin resistance and decreased hyperinsulinemia in muscle.^9^ In addition, voluntary running results in reduced tumor size in mice, due to increased recruitment and infiltration of natural killer (NK) immune cells,^7^ suggesting that exercise regulates cancer growth partially through improved immune responses. While the effects of exercise on cancer are multifactorial, the contribution of mechanical force, a principal component of physical activity, in regulating cancer progression is unclear.

While physical activity suppresses tumor growth and reduces cancer-related mortality, musculoskeletal complications arising from cancer treatments and cancer itself make exercise difficult at best, or physically dangerous at worst. The subsequent sedentary state perpetuates bone loss, and potentially the metastatic state,^10^ as the mechanical forces imparted through exercise are absent. Prior work demonstrates that the physical signals necessary to activate cellular responses need not be large, nor of long duration.^8,11^ As such, although low-intensity vibration is distinct from the high intensity mechanical input imparted through typical exercise regimens, introducing very low-magnitude mechanical vibration exogenously may provide the necessary benefits of mechanical input while avoiding the negative consequences of more strenuous forms of traditional exercise.

Low magnitude mechanical forces can be introduced to the musculoskeletal system through platforms that emit low-intensity vibration (LIV) signals, serving as an effective “exercise surrogate” by delivering mechanical input similar to that of exercise.^12^ LIV promotes proliferation and differentiation of mesenchymal progenitor cells.^13^ At the molecular level, LIV initiates a signaling cascade resulting in increased phosphorylation of focal adhesion kinase (FAK) and Akt, resulting in downstream activation of RhoA and formation of filamentous actin structures.^14^ The effects of LIV are additive, with a second bout of LIV enhancing FAK phosphorylation and F-actin contractility.^15^ The additive benefits observed with a second bout of mechanical stimuli have also been shown in animal^16^ and human studies,^17^ suggesting that proper dosing primes the cells to generate a more robust response with subsequent mechanical stimuli.

The mechanical compliance of tumor cells dictates cell behavior, where stiffness of the plasma membrane is inversely proportional to metastatic potential.^18^ Cells with decreased stiffness display increased migration and invasion, which is regulated by the organization of the actin cytoskeleton.^19^ Exogenous mechanical input enhances actin cytoskeletal structure,^14^ and work in non-cancerous cells demonstrates that the nucleus serves as a critical mechanosensory organ where direct connections between the nucleus and the cytoskeleton enable transmission of low-magnitude vibration.^15^ Attachment of the nucleus to the cytoskeleton is enabled by the LINC complex, containing Nesprin and Sun proteins, and may be a means by which physical activity influences the metastatic properties of cancer cells. Further, cells from human breast tumors have decreased expression of Nesprin and SUN,^20^ suggesting that the LINC complex may regulate tumorigenicity. In this work, we subjected human breast cancer cells to mechanical vibration and examined direct biochemical changes of the cancer cells, indirect paracrine signaling alterations, and the biophysical mechanisms enabling transmission of mechanical signals to breast cancer cells.

## Results

### LIV does not directly alter cell death but increases the susceptibility to Fas ligand-induced apoptosis

**Fig. 1:**
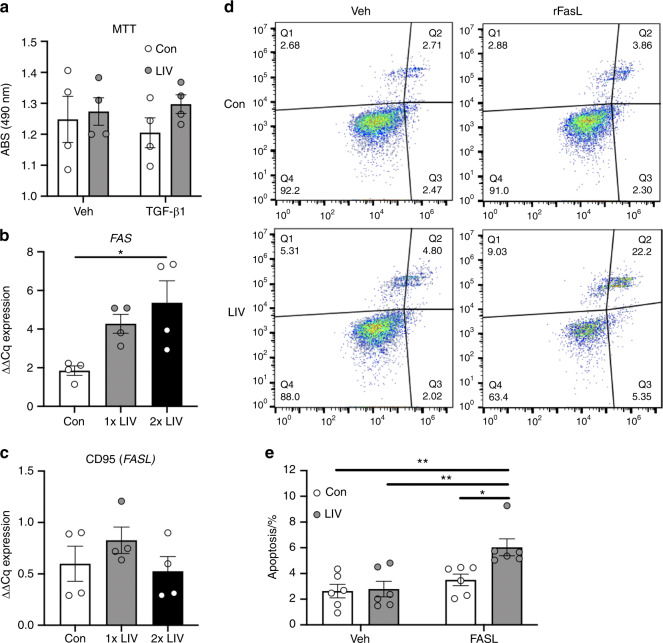
LIV increases susceptibility of FasL-mediated apoptosis. **a** MTT assay demonstrated no changes in cell viability, in the presence of absence of TGF-β1 following twice-daily LIV (2× LIV). Points represent individual measurements of biological replicates (*n* = 4). **b** Expression of *FAS* was quantified by qPCR and normalized to *GAPDH* (*n* = 4). Twice-daily LIV (2× LIV) significantly increased *FAS* expression, no significant difference was found with once-daily LIV (1x LIV). **c** qPCR analysis of *CD95*, normalized to *GAPDH* (*n* = 4). **d** Images obtained from flow cytometry of Annexin V-stained MDA-MB-231 cells. Quadrant #3 (Q3) represents early apoptosis. **e** Quantification of flow cytometry data from quadrant #3 following once- or twice-daily LIV, and treatment with PBS (veh) or recombinant Fas ligand (FASL). Data represent six independent biological replicates. One-way ANOVA (**b** and **c**) or Two-way ANOVA (**a** and **e**) *P*-values: *P < 0.05, **P < 0.01

Mechanical signals regulate cell death of several cancer types.^21^ To determine if direct application of LIV to breast cancer cells influences cell death, MDA-MB-231 human breast cancer cells were exposed to 20-min bouts of LIV (0.3 g, 90 Hz) once- or twice-daily for three days, in the presence or absence of TGF-β1. Control cells were placed on the vibration platform with no LIV transmission. Cell viability was assessed using the 3-(4,5-dimethylthiazol-2-yl)-2,5-diphenyltetrazolium bromide (MTT) assay. No changes in cell viability were observed with LIV or TGF-β1 treatment (Fig. 1a). mRNA expression of *FAS*, a cell membrane death receptor, was measured by quantitative polymerase chain reaction (qPCR). Once-daily LIV increased *FAS* expression by twofold compared to control (no LIV), but this change was not significant (Fig. 1b). Expression of *FAS* was significantly (*P* < 0.05) increased by threefold, compared to control, following twice-daily LIV (Fig. 1b). No significant differences were observed when direct comparisons of 1× and 2× treatments were made. Expression of *CD95*, the gene encoding for Fas ligand was not altered with LIV (Fig. 1c).

As twice-daily treatment with LIV-induced upregulation of *FAS*, we hypothesized that expression of the Fas death receptor may increase the susceptibility to Fas ligand-mediated apoptosis. As such, MDA-MB-231 cells were exposed to twice-daily LIV or placed on LIV platforms with no signal transmitted (control) and treated with recombinant Fas ligand (75 ng·mL^−1^) for 24 h prior to staining with Annexin V and subsequent sorting by flow cytometry (Fig. 1d). No changes in apoptosis were observed following LIV in vehicle-treated cells (Fig. 1e). Treatment with Fas ligand did not alter apoptosis in control cells; however, addition of Fas ligand to cells treated with LIV increased (*P* < 0.05) the percent of apoptotic cells compared to control cells by ~2-fold (Fig. 1e). These data demonstrate that application of LIV does not directly induce cell death, but that MDA-MB-231 cells are more susceptible to Fas ligand-mediated apoptosis following exposure to mechanical vibration.

### Low magnitude mechanical vibration suppress invasion

**Fig. 2:**
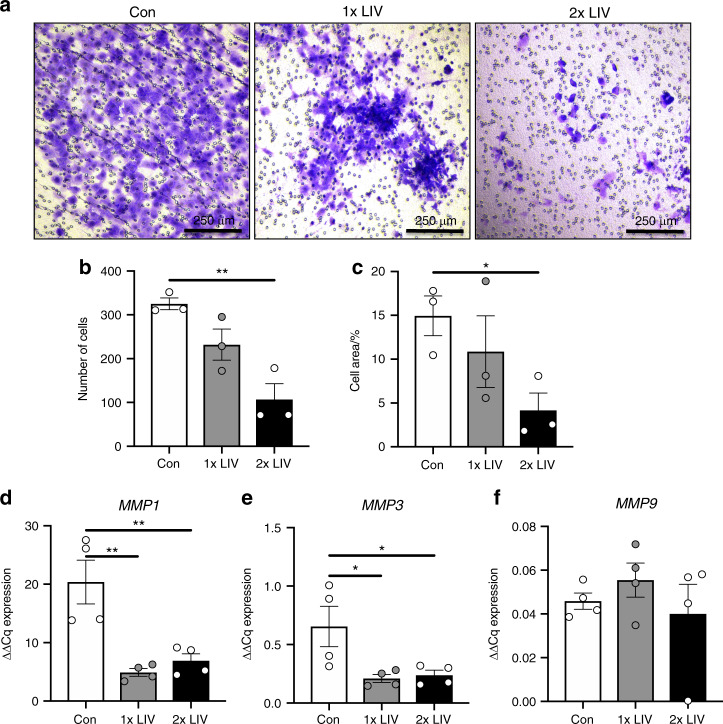
LIV suppresses invasion of MDA-MB-231 cells. **a** Representative images showing crystal violet staining (purple) of MDA-MB-231 cells exposed to once- (1× LIV) or twice-daily (2× LIV) LIV that have invaded through Matrigel^®^ and penetrated through the trans-well membrane. Images are representative of three biological replicates. **b** Quantification of crystal violet stained cells that invaded through the trans-well membrane. Compared to non-vibrated controls (Con) once-daily LIV (1× LIV) induced no significant change, while twice-daily LIV (2× LIV) resulted in a significant reduction in invasion (*n* = 3). **c** Invasion through the trans-well membrane was also quantified by measuring the total area of crystal violet stained cells, relative to the total area of the membrane (*n* = 3). Similar results were found with a significant reduction in invasion following twice-daily LIV (2× LIV). **d** Expression of matrix metalloproteinase 1 (*MMP1*) mRNA (*n* = 4). **e** Expression of *MMP3* mRNA by qPCR (*n* = 4). **f** Expression of *MMP9* mRNA by qPCR (*n* = 4). One-way ANOVA *P*-values: *P < 0.05, **P < 0.01

Extravasation and subsequent metastasis of cancer cells requires invasion through matrix-dense borders. To determine if exogenously applied LIV influenced the ability of MDA-MB-231 cells to invade through ECM, trans-well invasion assays were performed. Cells were treated with LIV for 20 min per bout, once- or twice-daily for three days. Cells then were trypsinized and seeded onto trans-well membranes containing Matrigel^®^ and visualized using crystal violet (Fig. 2a). Once-daily LIV induced a 28.6% reduction in invasion; however, cells exposed to LIV twice-daily had a significant (*P* < 0.01) 67.1% reduction (Fig. 2b). Direct comparison of once-daily to twice-daily LIV reveals a 54% decrease in cell invasion with twice-daily LIV compared to the once-daily treatment which trended toward significant (*P* = 0.078). The area of cells that invaded through the trans-well membrane was also quantified, showing a significant (*P* < 0.05) reduction in invasion following twice-daily LIV, while there was no significant difference between 1× and 2× treatments when compared directly (Fig. 2c).

As invasion through the ECM requires matrix metalloproteinases (MMPs),^22^ MMP mRNA levels were quantified by qPCR. *MMP1* expression was decreased by 75.9% (*P* < 0.01) following once-daily LIV and by 66.1% (*P* < 0.01) with twice-daily LIV (Fig. 2d). Expression of *MMP3* was reduced by 67.9% (*P* < 0.05) following once-daily LIV and by 63.5% (*P* < 0.05) with twice-daily LIV (Fig. 2e). No changes in *MMP9* expression were observed with once- or twice-daily LIV (Fig. 2f).

### Exposure of breast cancer cells to LIV impairs osteoclastogenesis

**Fig. 3:**
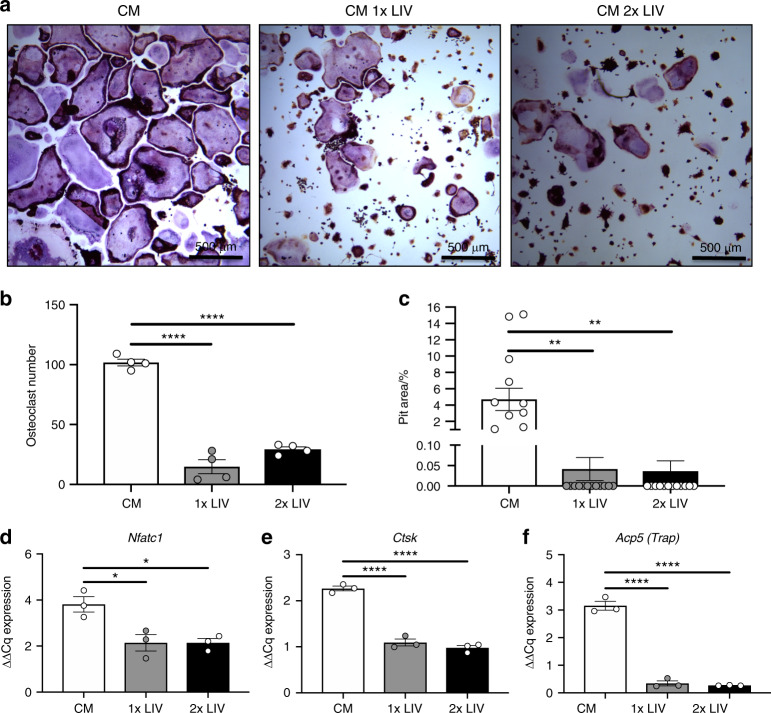
Mechanical stimulation of breast cancer cells releases factors that suppress osteoclastogenesis. **a** Representative images of murine RAW 264.7 cells stained with TRAP after addition of conditioned media from MDA-MB-231 cells not receiving LIV (CM), receiving once-daily LIV (CM 1× LIV) or twice-daily LIV (CM 2× LIV). Images are representative of four biological replicates. **b** Quantification of the number of osteoclasts per area, following treatment with CM from MDA-MB-231 cells. Cells were counted as osteoclasts if they contained positive staining for TRAP (purple) and contained ≥3 nuclei (*n* = 4). **c** RAW 264.7 cells were plated on Osteoassay wells containing hydroxyapatite and exposed to conditioned media from MDA-MB-231 cells, as described above. The area of hydroxyapatite that was resorbed away from the dish was quantified using ImageJ and normalized to the total area (*n* = 10). **d** Quantification of *Nfatc1* mRNA from differentiated RAW 264.7 cells following addition of CM from MDA-MB-231 cells (*n* = 3). Quantification of mRNA from differentiated RAW 264.7 cells following addition of CM from MDA-MB-231 cells for genes that regulate osteoclast differentiation including **d** nuclear factor of activated T-cells (*Nfatc1*), **e** cathepsin-K (*Ctsk*), **f** tartrate resistant acid phosphatase (*Acp5*). Data for each graph represent three biological replicates. One-way ANOVA *P*-values: *P < 0.05, **P < 0.01, ****P < 0.000 1

Breast cancer readily metastasizes to bone, resulting in osteolysis through increased osteoclast formation.^23^ Tumor-mediated activation of osteoclasts results in an enhanced state of bone resorption and release of matrix-derived growth factors, further stimulating tumor cell invasion and growth.^23^ LIV restricts cancer-induced bone loss,^24,25^ possibly the result of reduced secretion of pro-osteolytic factors from the tumors themselves. To determine if direct application of LIV to cancer cells impairs osteoclastogenesis, MDA-MB-231 cells were exposed to LIV once- or twice-daily, as described. Conditioned media (CM) was collected from MDA-MB-231 cultures 3 h after the last LIV treatment. Murine RAW 264.7 macrophages were exposed to media from control or LIV-treated MDA-MB-231 cells and stained for tartrate resistant acid phosphatase (TRAP). RAW 264.7 cells exposed to media from control MDA-MB-231 cells readily differentiated into multinucleated (>3 nuclei), TRAP positive, osteoclasts; whereas treatment with LIV, once- or twice-daily restricted osteoclast formation (Fig. 3a). Quantification of TRAP stained cultures demonstrated a significant (*P* < 0.000 1) reduction in the number of multinucleated cells (≥3 nuclei) following exposure of RAW 264.7 cells to CM from MDA-MB-231 cells vibrated once- (85.5%) or twice-daily (71.2%) LIV (Fig. 3b). No changes were observed when directly comparing 1× to 2× treatments.

To assess the activity of osteoclasts exposed to CM from MDA-MB-231 cells, RAW 264.7 cells were seeded to osteoassay wells (Corning, Corning NY) containing hydroxyapatite. Cells were treated with CM from MDA-MB-231 cells exposed to once- or twice-daily LIV. RAW 264.7 cells exposed to CM from once- and twice-daily LIV-treated MDA-MB-231 cells reduced (*P* < 0.01) the resorption pit area by 99.1% and 99.2% respectively (Fig. 3c). These data demonstrate that mechanically stimulating MDA-MB-231 breast cancer cells reduces their ability to support both osteoclast formation and activity.

Osteoclast formation requires the transcription factor nuclear factor of activated T cells 1 (*NFATC1*) and is accompanied by the subsequent production of genes including cathepsin K (Ctsk) and *Trap*.^26^ To determine how CM from LIV-treated breast cancer cells reduced osteoclast formation, RNA from RAW 264.7 cells was examined by qPCR following exposure to CM. Exposure of RAW 264.7 cells to CM from MDA-MB-231 cells treated with once- or twice-daily LIV reduced *Nfatc1* expression by 49.6% and 44% respectively (*P* < 0.05) (Fig. 3d). Expression of *Ctsk* was reduced (*P* < 0.01) in cells treated with CM from once- (47.2%) and twice-daily (59.3%) treated MDA-MB-231 cells (Fig. 3e). *Trap* (*Acp5*) expression was also reduced (*P* < 0.05) following treatment with CM from once- and twice-daily LIV-treated MDA-MB-231 cells, with reductions of 91.6% and 92% respectively (Fig. 3f). These data suggest that the reductions in osteoclast number, following exposure to CM from mechanically stimulated breast cancer cells, are associated with suppression of osteoclast regulatory genes, including *Nfatc1*, *Ctsk*, and *Acp5*.

### LIV suppresses expression of factors that promote osteolysis

Within the bone microenvironment, cancer cells produce factors that perpetuate osteoclast formation and subsequent osteolysis. As we found that CM from human breast cancer cells, exposed to LIV, suppressed osteoclast formation, we next sought to quantify expression of factors produced by MDA-MB-231 cells that promote osteolysis, following LIV. As TGF-β enhances the metastatic phenotype of cancer cells, and increases production of osteolytic factors,^27^ MDA-MB-231 were treated with TGF-β1 (5 ng·mL^−1^, w/v) or PBS (veh) as a control, prior to LIV. Exposure of MDA-MB-231 cells to LIV for 20 min once per day for 3 days resulted in a 60.7% decrease of parathyroid hormone like hormone (*PTHLH*) expression (*P* < 0.01) in the absence of TGF-β1 and a decrease of 29.7% (*P* < 0.05) in the presence of TGF-β1 (Fig. S1a). There were no changes in expression of connective tissue growth factor (*CTGF*), *CXCR4*, or interleukin 11 (*IL-11*) following once-daily LIV (Fig. S1b–d).

**Fig. 4:**
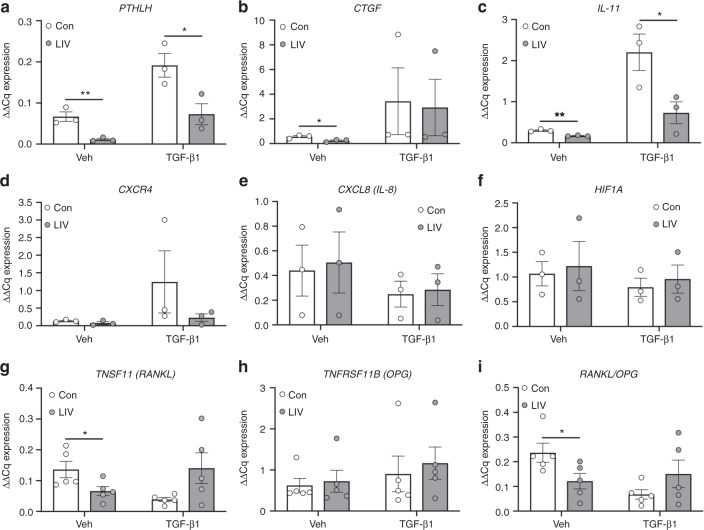
Low-magnitude mechanical forces suppress expression of osteolytic genes. MDA-MB-231 cells were treated with PBS (Veh) or TGF-β1 and exposed to non-vibration control conditions (Con) or LIV twice-daily for 3 days. qPCR analyses were normalized to *GAPDH*. Genes surveyed included **a** parathyroid hormone-related protein (*PTHLH*) (*n* = 3), **b** connective tissue growth factor (*CTGF*) (*n* = 3), **c** interleukin 11 (*IL-11*) (*n* = 3), **d** C-X-C chemokine receptor type 4 (*CXCR4*) (*n* = 3), **e** interleukin 8 (*CXCL8*) (*n* = 3), **f** hypoxia-inducible factor 1-alpha (*HIF1A*) (*n* = 3), **g** receptor activator of nuclear factor kappa-B ligand (TNFSF11, *RANKL*) (*n* = 5), and **h** osteoprotegerin (*TNFRSF11B, OPG*) (*n* = 5). **i** Quantification of the ratio of *RANKL* to *OPG* mRNA (*n* = 5). Multiple *t*-test *P*-values: *P < 0.05, **P < 0.01

Previous work in non-tumor cells showed that twice-daily LIV resulted in enhanced anabolic effects compared to once-daily treatment,^28^ likely due to a “priming” effect following the first bout of mechanical force. To determine if a similar priming effect occurs in breast cancer cell expression of pro-osteolytic/metastatic genes in MDA-MB-231 cells were also quantified following twice-daily LIV treatment for 3 days. Treatment with LIV twice a day resulted in reduced *PTHLH* expression in both the absence (83.1%, *P* < 0.01) and presence (62%, *P* < 0.05) of TGF-β1 (Fig. 4a). LIV induced a reduction of *CTGF* of 57.6% (*P* < 0.05) in the absence of TGF-β1; however, no differences were found in the presence of TGF-β1 (Fig. 4b). Expression of *IL-11* mRNA was reduced both in the presence (43.3%, *P* < 0.01) and absence (66.9%, *P* < 0.05) of TGF-β1 following twice-daily LIV. Levels of C-X-C chemokine receptor type 4 (*CXCR4*), interleukin 8 (*CXCL8*), and hypoxia-inducible factor 1-alpha (*HIF1A*) were also quantified and no differences were observed with LIV, regardless of the addition of TGF-β1 (Fig. 4d–f).

Osteoclast formation requires binding of receptor activator of nuclear factor kappa-B ligand (RANKL) to the RANK receptor. While thought to be predominantly produced by osteoblasts, RANKL is also secreted by breast cancer cells.^29–31^ In contrast to RANKL, osteoprotegerin (OPG) suppresses osteoclast formation by acting as a decoy receptor for RANKL.^31^ Exposure of MDA-MB-231 cells to twice-daily LIV reduced *RANKL* expression by 51.3% (*P* < 0.05). When treated with TGF-β1, a non-significant increase of 3.6-fold in RANKL was found following LIV treatment (Fig. 4g). *OPG* expression was not altered with LIV in the presence or absence of TGF-β1 (Fig. 4h); however, the ratio of *RANKL* to *OPG* expression was reduced by 48.7% (*P* < 0.05) in the absence of TGF-β1 and not significantly altered in the presence of TGF-β1 (Fig. 4i). These results demonstrate that LIV suppresses the production of genes in breast cancer cells that influence osteoclast formation.

Additional analyses were completed to directly compare expression levels following once- and twice-daily LIV treatments. Twice-daily LIV suppressed *PTHLH* in the presence of TGF-β1, but no change was observed in the absence of TGF-β1 (Fig. S2a). No changes in *CTGF* were observed between 1× and 2× LIV (Fig. S2b). Twice-daily LIV suppressed *CXCR4* in the presence of TGF-β1, but no difference was observed in the absence of TGF-β1 (Fig. S2c). Significant reductions in *IL-11* expression were observed in twice-daily treated MDA-MB-231 cells compared to once-daily, but only in the absence of TGF-β1 (Fig. S2d). Expression of TNSF11 (RANKL) was significantly reduced with twice-daily treatment (Fig. S2e), but no changes were observed between 1× and 2× for TNFRSF11B (OPG) (Fig. S2f) or for the RANKL/OPG ratio (Fig. S2g).

### Low magnitude mechanical vibration increases expression of LINC complex genes

**Fig. 5:**
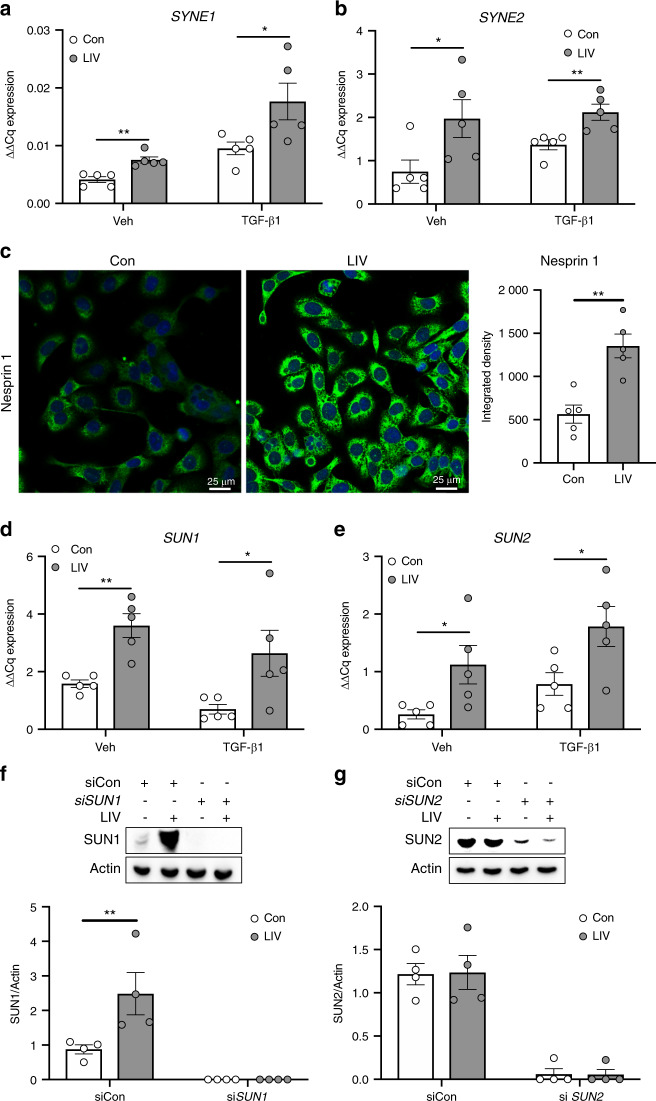
LIV upregulates production of LINC complex components. qPCR analyses of (**a**) nesprin 1 (*SYNE1*) and (**b**) nesprin 2 (*SYNE2*) mRNA from MDA-MB-231 cells treated with PBS (Veh) or TGF-β1 and exposed to non-vibration control conditions (Con) or LIV twice-daily for 3 days. qPCR analyses were normalized to *GAPDH* (*n* = 5). **c** Representative images of immunocytochemistry staining of nesprin 1 in MDA-MB-231 cells treated twice-daily with LIV showing increased nesprin 1 signal following LIV. Images are representative of three biological replicates. qPCR analyses of (**d**) sun 1 (*SUN1*) and (**e**) sun 2 (*SUN2*) mRNA from MDA-MB-231 cells treated with PBS (Veh) or TGF-β1 and exposed to non-vibration control conditions (Con) or LIV twice-daily for 3 days. qPCR analyses were normalized to *GAPDH* (*n* = 5). Western blots of whole cell lysates (20 μg per lane) from MDA-MB-231 cells transfected with a control siRNA (siCon) or (**f**) siRNA targeting *SUN1* (si*SUN1*) or (**g**) siRNA targeting *SUN2* and exposed to non-vibration control conditions (Con) or LIV twice-daily for 3 days. PVDF membranes were blotted with an antibody recognizing SUN1 and β-actin as a loading control. Densitometry was measured using ImageJ and normalized to β-actin. Western blot images are representative of four biological replicates. Uncropped images shown in supplementary fig. 5. Multiple *t*-test *P*-values: *P < 0.05, **P < 0.01

High magnitude mechanical forces, including membrane deformation and fluid shear stress, activate intracellular signaling through focal adhesions (FAs) at the plasma membrane.^14^ In contrast, low-magnitude vibrational forces (<1 g) do not induce appreciable fluid shear forces, nor deformation of the plasma membrane,^32–34^ suggesting that low-magnitude vibration signals are not transmitted through the cell membrane. Recent work showed that the nucleus acts as a critical mechanosensory organelle and that transmission of low-magnitude mechanical vibration requires connections between the nucleus and the actin cytoskeleton, which are mediated by the LINC complex.^15^ To determine if LIV influenced expression of LINC complex genes, Nesprin and Sun, MDA-MB-231 cells were treated with recombinant TGF-β1 or PBS (veh) and subjected to LIV twice daily for three days. Exposure to LIV, in the absence or presence of TGF-β1 resulted in a 1.82-fold (*P* < 0.01) and 1.84-fold (*P* < 0.05) increase in *SYNE1*, the gene encoding Nesprin 1, expression respectively (Fig. 5a). *SYNE2* (Nesprin 2) mRNA was increased by 2.63-fold (*P* < 0.05) in the absence and 1.54-fold (*P* < 0.01) in the presence of TGF-β1 (Fig. 5b). The molecular weight of Nesprin1 (>1 000 kDa) presents challenges for resolving this large protein via Western blotting, thus immunocytochemistry was performed. Cells exposed to 3 days of twice-daily LIV had noticeably more fluorescent labeling compared to non-vibrated cells (Fig. 5c). Although fluorescent signal from immunocytochemistry assays is non-stoichiometric, quantification of the fluorescent intensity displayed a 2.4-fold increase (*P* < 0.01) of Nesprin1 fluorescence signal compared to non-vibrated controls (Fig. 5c).

Transcript levels of *SUN1* (Fig. 5d) and *SUN2* (Fig. 5e) were increased by ~2–4 fold following LIV both in the absence and presence of TGFβ. Protein expression of SUN1 and SUN2 was assessed by Western blotting. SUN1 was increased by 2.8-fold (*P* < 0.01) following LIV (Fig. 5f), while SUN2 was not altered (Fig. 5g). Treatment with siRNA targeting *SUN1* (Fig. 5f) or *SUN2* (Fig. 5g) knocked down each protein to nearly undetectable levels. Full size, non-cropped blots are shown in Fig. S3. These findings demonstrate that LIV enhances transcript expression of *SYNE1*, *SYNE2*, *SUN1*, and *SUN2* while increasing protein expression of both Nesprin1 and SUN1 in human breast cancer cells.

### LIV-induced suppression of osteolytic factors requires the LINC complex

**Fig. 6:**
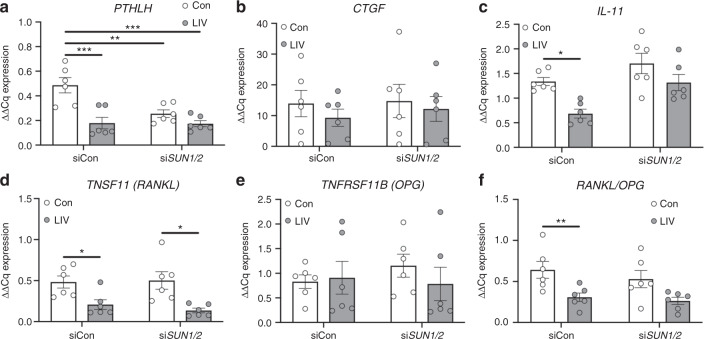
SUN1 and SUN2 are necessary for mechanical repression of osteolytic genes. MDA-MB-231 cells were treated with control siRNA sequences (siCon) or siRNA targeting *SUN1* and *SUN2* and exposed to non-vibration control conditions (Con) or LIV twice-daily for 3 days. qPCR analyses were normalized to *GAPDH*. Genes surveyed included (**a**) *PTHLH*, (**b**) *CTGF*, (**c**) *IL-11*, (**d**) TNFSF11 (*RANKL*), and (**e**) *TNFRSF11B* (*OPG*). **i** Quantification of the ratio of *RANKL* to *OPG* mRNA. All graphs represent six biological replicates (*n* = 6). Two-way ANOVA *P*-values: *P < 0.05, **P < 0.01, ***P < 0.001

The increase in expression of LINC complex genes, following LIV, suggested that low-magnitude mechanical forces enhance LINC connectivity, possibly accounting for the downregulation of factors that support osteoclast formation following LIV. As such, MDA-MB-231 cells were transfected with a control siRNA, of similar sequence as the targeting siRNA with variation of critical nucleotides, or siRNA targeting both *SUN1* and *SUN2*, to physically disconnect the LINC complex from the actin cytoskeleton. siRNA targeting both transcripts simultaneously was used as *SUN1* and *SUN2* have redundant functions for binding actin.^35^ Furthermore, while LIV resulted in increases of both SYNE and SUN genes (Fig. 5), knockdown of SUN1/2 was chosen as the strategy to functionally disconnect the nucleus from the cytoskeleton as verification of knockdown is technically less challenging than SYNE and previous work has shown that disruption of SUN is sufficient to impair nucleocytoskeletal connectivity.^35–38^ Cells were exposed to LIV twice daily for 3 days, as described. While expression of *PTHLH* was significantly reduced (60%, *P* < 0.01) in cells treated with a control siRNA following LIV, knockdown of *SUN1* and *SUN2* (denoted as *SUN1/2*) resulted in the inability of LIV to suppress *PTHLH* (Fig. 6a). LIV treatment resulted in no changes in expression of *CTGF* with control siRNA or siRNA targeting *SUN1/2* (Fig. 6b). LIV-mediated downregulation of *IL11* expression (48.5%, *P* < 0.05) was mitigated following knockdown of *SUN1/2* (Fig. 6c). Expression of *TNSF11* (*RANKL*) was suppressed by LIV with intact *SUN1/2* expression (56.6%, *P* < 0.05) and following siRNA-mediated *SUN1/2* knockdown (73.7%, *P* < 0.05) (Fig. 6d). LIV had no impact on expression of *TNFRSF11B* (*OPG*) (Fig. 6e); however, when the *RANKL/OPG* ratio was evaluated, the suppression observed with LIV treatment (54%, *P* < 0.05) was negated following knockdown of *SUN1/2*.

MCF-7 human breast cancer cells were also subjected to LIV, following transfection with control siRNA or siRNA targeting *SUN1/2*. Similar to MDA-MB-231 cells, LIV resulted in decreased expression of *IL-11*, *TNSF11* and the ratio of *TNSF11* to *TNFRSF11B* (*RANKL/OPG*) (Fig. S2). Knockdown of *SUN1/2* negated the ability of LIV to suppress expression of these genes in MCF-7 cells. In control cells (no LIV) knockdown of *SUN1/2* resulted in increased *CXCR4* (Fig. S4). These data demonstrate that LIV-mediated downregulation of osteoclastic factors produced by breast cancer cells require an intact LINC complex.

### LIV regulates secretion of osteolytic factors from MDA-MB-231 cells

To determine if protein production and secretion of factors that promote osteolysis are influenced by LIV, CM was collected from MDA-MB-231 cells treated with control siRNA or siRNA targeting *SUN1/2* following 3 days of twice-daily LIV. No differences were observed in PTHrP production following LIV with control siRNA or *SUN1/2* siRNA (Fig. S5a). Consistent with transcript levels, secretion of IL11 was reduced following LIV (46.2%, *P* < 0.05), while knockdown of *SUN1/2* resulted in increased IL11 production with LIV (2.8-fold, *P* < 0.05) (Fig. S5b). Secretion of RANKL was reduced by LIV (62.1%, *P* < 0.5) in cells treated with control siRNA; however, knockdown of *SUN1/2* resulted in a non-significant increase in RANKL (1.9-fold) (Fig. S5c). While there were no changes in OPG secretion with control siRNA or si*SUN1/2* (Fig. S5d), the ratio of RANKL to OPG was decreased in cells transfected with control siRNA (43.2%, *P* < 0.05) but increased (2.1-fold, *P* > 0.5) following *SUN1/2* knockdown (Fig. S5e). These data indicate that exposing breast cancer cells to twice-daily LIV reduces secretion of factors that support osteoclastogenesis, and the LINC complex is necessary for these changes.

### LIV-mediated suppression of invasion and osteoclastogenesis requires the LINC complex

**Fig. 7:**
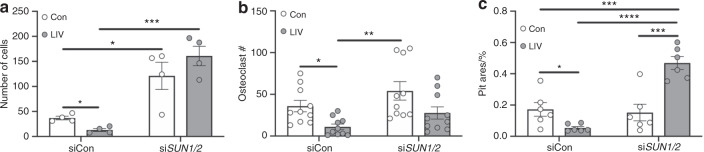
Mechanical suppression of breast cancer cell invasion and signaling to osteoclasts requires SUN1 and SUN2. **a** Quantification of cells (normalized to total area) invading through trans-well membrane under control (Con, no LIV) or twice-daily LIV (LIV) conditions. Cells were transfected with control siRNA oligos (siCon) or with siRNAs targeting both *SUN1* and *SUN2* (si*SUN1/2*). Data are representative of four biological replicates. Representative images are shown in Supplementary Fig. 6. **b** Quantification of the number of osteoclasts following exposure of RAW 264.7 cells to conditioned media from MDA-MB-231 cells that received LIV twice-daily for 3 days and transfected with control siRNA sequences (siCon) or siRNAs targeting *SUN1* and *SUN2* (si*SUN1/2*) prior to LIV. Data were compiled from ten biological replicates and representative images are shown in Supplementary Fig. 8. **c** Osteoclast resorption, as measured by the area (%) of hydroxyapatite resorbed by differentiated RAW 264.7 cells on Osteoassay plates following the addition of conditioned media from MDA-MB-231 conditioned media. MDA-MB-231 cells were transfected with siRNA sequences targeting *SUN1* and *SUN2* (si*SUN1/2*), as mentioned above, and exposed to twice-daily LIV, or non-LIV control conditions (Con). Data compiled from six biological replicates and measured using ImageJ. Two-way ANOVA *P*-values: *P < 0.05, **P < 0.01, ***P < 0.001, ****P < 0.000 1

To determine if the LINC complex is necessary for the functional effects of LIV on invasion of MDA-MB-231 cells, and the ability of secreted factors to support osteoclast formation, *SUN1/2* were knocked down using siRNA, and cells were treated with LIV as described. The 66.5% reduction in invasion of MDA-MB-231 cells through Matrigel (*P* < 0.05) in cells treated with a control siRNA was negated following knockdown of *SUN1/2*, where no differences in invasion were observed (Figs. 7a and S6). Similarly, MCF-7 cells treated with LIV displayed a 41.5% reduction (*P* < 0.05) in invasion, while LIV had no effect following knockdown of *SUN1/2*. In control cells (no LIV), knockdown of *SUN1/2* resulted in a 1.9-fold increase (*P* < 0.05) in invasion (Fig. S7a).

Osteoclast formation of RAW 264.7 macrophages was evaluated following treatment with CM from non-vibrated and vibrated MDA-MB-231 or MCF-7 cells. CM from LIV-treated cells resulted in a reduction in multinucleated (>3 nuclei) osteoclasts for both MDA-MB-231 (Figs. 7b and S8, 69.3%, *P* < 0.05) and MCF-7 (Fig. S7b, 16.3%, *P* < 0.05); whereas CM from both cell lines, following knockdown of *SUN1/2*, resulted in no differences in formation of osteoclasts.

To assess resorption activity, following exposure to CM from MDA-MB-231 cells, RAW 264.7 macrophages were seeded on Osteo Assay wells. Cells exposed to CM from LIV-treated MDA-MB-231 cells, transfected with control siRNA, had a 68.7% (*P* < 0.05) decrease in resorption area compared to non-vibrated controls (Fig. 7c). In contrast, addition of CM from cells exposed to LIV, but transfected with siRNA targeting *SUN1 and SUN2*, resulted in a 3.3-fold increase in resorption (*P* < 0.000 1). These data demonstrate that knockdown of Sun1 and Sun2 are essential for the ability of LIV to regulate breast cancer cell invasion and support of osteoclast formation and activity.

### The LINC nuclear complex is required for mechanically induced cellular stiffness

**Fig. 8:**
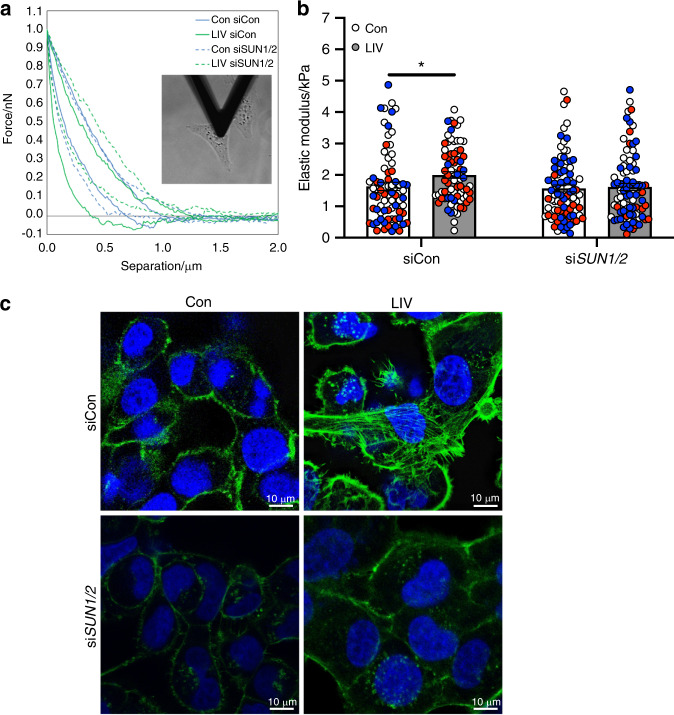
Enhanced cell stiffness, induced by low-magnitude mechanical forces, requires the LINC complex. **a** Image showing the cantilever of the AFM resting over the nucleus of a single MDA-MB-231 cell and representative plot showing forces generated during the approach and retraction of the borosilicate probe (attached to the cantilever) as a function of distance. **b** Graph showing quantification of the elastic modulus (E, kPa). Individual cells from three biological replicate assays were tested. The number of cells tested for each group are as follows. Con/siCon: *n* = 74, LIV/siCon: *n* = 76, Con/si*SUN1/2*: *n* = 85, LIV/si*SUN1/2*: *n* = 85. Each data point is from AFM measurements of a single cell. Data points from the same biological replicate are designated by color (red, white, blue). **c** Representative images of MDA-MB-231 cells exposed to non-vibration control conditions (Con), twice-daily LIV, and either control siRNAs or siRNA oligos targeting *SUN1* and *SUN2* (si*SUN1/2*). For each condition, cells were fixed following a 3-h rest period after the last LIV bout and incubated with Phalloidin-conjugated Alexa-Fluor 488 and Dapi to stain for filamentous actin (green) and nuclei (blue) respectively. Images are representative of three biological replicates. Statistical analyses calculated using two-way ANOVA, followed by two-stage linear step-up procedure of Benjamini, Krieger, and Yekutieli to control for false discovery rate

The mechanical integrity of cancer cells is inversely proportional to metastatic potential.^19^ We hypothesized that the suppression of invasion and decreases in osteoclastogenesis, observed following LIV, may be the result of increased cell membrane stiffness. To quantify membrane stiffness, MDA-MB-231 cells were exposed to LIV twice-daily for 3 days, or to non-vibrated conditions. Cells were transfected with either control siRNAs or siRNAs targeting *SUN1* and *SUN2*, as described. Cellular stiffness (elastic modulus) was measured by atomic force microscopy, as in Fig. 8a. In cells treated with control siRNA, LIV increased cellular stiffness (1.2-fold, *P* < 0.05), while no differences were found in cells after knockdown of *SUN1/2* (Fig. 8b).

As part of the LINC complex, SUN proteins enable connection between the actin cytoskeleton and the nucleus. Low magnitude mechanical forces enhance actin cytoskeletal structure, which may account for the increased cellular stiffness. Exposure of MDA-MB-231 (Fig. 8c) and MCF-7 (Fig. S9) cells to LIV twice daily for 3 days resulted in increased actin cytoskeleton stress fiber formation, as stained by Alexa 488-conjugated phalloidin, compared to non-vibrated controls. The increased cytoskeletal structure observed with LIV was negated with knockdown of *SUN1/2*, resulting in phalloidin signal similar to that of non-LIV controls in both MDA-MB-231 (Fig. 8c) and MCF-7 (Fig. S9) cells.

## Discussion

A variety of chemical, hormonal, and physical cues regulate cell behavior; many of which are influenced by exercise. Mechanical signals are a principal component of exercise and are not only instrumental in governing adaptation of musculoskeletal tissues, but also influence cancer cell behavior including invasion,^39^ division,^40^ migration,^41^ and metastasis.^42^ Here we demonstrate that very low-magnitude mechanical signals, introduced in the form of low-intensity vibration regulates invasive, apoptotic, and osteoclastogenic properties of human breast cancer cells through biochemical alterations of the cancer cells, via paracrine signaling between tumor cells and osteoclasts, and via biophysical changes in the cancer cells in response to the exogenous mechanical vibration. Our data show that LIV directly influences human breast cancer cells by suppressing invasion and increasing the susceptibility to apoptosis. Application of mechanical vibration also influenced paracrine signaling, resulting in suppressed osteoclast differentiation and lytic activity. Furthermore, LIV altered the biophysical properties of cancer cells, resulting in increased membrane stiffness, where force transmission required connection between the nucleus and the actin cytoskeleton, mediated by the LINC complex.

Exercise is an effective form of introducing exogenous mechanical signals to promote musculoskeletal anabolism; ^43^ however, common forms of weight-bearing exercise such as running pose challenges to cancer patients, who are already debilitated due to their illness and subsequent treatments. This can lead to injuries, falls, and fractures, the very thing exercise was intended to prevent. LIV introduces the necessary mechanical input to maintain musculoskeletal health,^11,44,45^ yet correct dosing is essential. In non-cancerous cells, delivery of two bouts of mechanical input separated by a rest period of 3 h induced greater anabolic signaling than when the mechanical stimulation was delivered for the same overall time duration but with no rest period.^28^ These findings mimic responses in mice where incorporation of a refractory period between bouts of mechanical stimulation diminished obesity-induced adipose accumulation.^16^ In this study, incorporation of a refractory period between bouts of mechanical vibration resulted in greater effects compared to once-daily treatment for some endpoints but not all. Twice-daily LIV resulted in significantly decreased invasion compared to non-vibrated cells, while there was no change compared to controls for once-daily LIV. However, there was no direct difference between once- and twice-daily treatments. In contrast to the invasion assays, CM from both once- and twice-daily LIV-treated MDA-MB-231 cells resulted in significant reductions in osteoclast formation. In addition, qPCR analyses showed that twice-daily LIV resulted in significantly lower expression of some genes, including *PTHLH*, *IL-11*, and *TNSF11 (RANKL)* compared to once-daily LIV; however, this was not the case for all genes analyzed. Regulation of metastatic potential and signaling of cancer cells to osteoclasts is complex, and likely involves the coalescence of many signaling cascades. As such, while twice-daily LIV is not definitively better for all assays examined, our data show that it is more potent for some endpoints and thus may be critical to the overall response of LIV in vitro and in vivo. A recent randomized clinical trial in childhood cancer survivors demonstrated that twice-daily LIV treatment, seven days per week for one year resulted in up to 11% greater BMD.^17^ While the exact pathways and dosing parameters require additional investigation, these clinical data, along with the results from the work presented here and other recent animal and cell^46^ studies provide important insight for optimal dosing of mechanical forces in clinical settings.

Mechanical signals influence tumor cell death. In one study, fluid shear increased apoptosis in several tumor cell types, while no negative effects of shear were found in non-tumor cells.^21^ In another study, daily application of LIV increased apoptosis of MDA-MB-231 cells.^47^ We did not observe increased cell death directly following LIV; however, LIV increased expression of the membrane death receptor *FAS*, resulting in increased Fas ligand-mediated apoptosis following LIV. As immune cells, including natural killer (NK) cells and T cells,^48^ produce Fas-ligand, our data suggest that LIV renders breast cancer cells more susceptible to immune-mediated apoptosis. In previous work, voluntary running decreased tumor size in mice, an effect that was linked to increased T and NK cell tumor infiltration.^7^ Clearing of NK cells from mice negated the effect of exercise.^7^ Our data demonstrate that introduction of exogenous mechanical signals regulates susceptibility of immune-mediated responses in breast cancer cells, a possible explanation for altered tumor size following exercise in mice.

In addition to the effects of mechanical vibration on cell survival, we found that application of LIV reduced breast cancer cell invasion in vitro. These changes were accompanied by reductions in MMPs. As MMPs are associated with increased breast cancer metastases,^49,50^ our data suggest that mechanical vibration suppresses breast cancer metastasis by reducing the ability of tumor cells to invade through matrix in vitro.

The skeleton is a frequent site of breast cancer metastasis.^51^ Once localized to bone, breast cancer cells release factors, including PTHrP,^52^ IL-11,^53^ CTGF,^54^ and RANKL,^55^ which increase osteoclast activity, leading to osteolysis and tumor progression.^23,56^ As such, we examined the ability of mechanical vibration to regulate release of paracrine signals from breast cancer cells. Here we show that direct application of LIV to human breast cancer cells reduced expression and secretion of RANKL and IL-11. Furthermore, when exposed to CM from LIV-treated breast cancer cells, osteoclast precursors had reduced differentiation and resorption activity. Previous work in murine models of ovarian cancer^24^ and myeloma^25^ report that LIV diminished the deleterious effects of cancer metastasis on bone structure, specifically by ameliorating cancer-induced osteoclast resorption of trabecular surfaces.^25^ The reduced trabecular erosion may be the result of direct effects of LIV on osteoclasts, or through LIV-mediated alterations in paracrine signaling of cancer cells to osteoclasts. Data supporting the direct effects of LIV on osteoclast differentiation are conflicting,^57,58^ where results may be influenced by the regimen of mechanical vibration. Our data demonstrate that direct application of LIV to breast cancer cells impairs paracrine signaling necessary for osteoclast activation, an outcome that may be linked to the biophysical responses of the tumor cells.

Mechanical properties of tissues influence behavior. Tumors are stiffer than healthy tissue, due to altered matrix composition.^59,60^ In contrast, the plasma membrane of metastatic cells is less stiff than non-metastatic cells.^19,61,62^ In addition, cell stiffness is increased by enhanced actin cytoskeletal structure, which is associated with decreased metastatic potential.^63^ Application of mechanical force increases actin cytoskeletal structure,^46^ where increased filamentous actin is frequently interpreted as enhanced cellular stiffness. Using AFM, we found that LIV increased the stiffness of human breast cancer cells in vitro. This effect was negated following knockdown of *SUN1/2*. These results correlated well with changes in filamentous actin where, in contrast to control cells, LIV resulted in an increase of structured actin stress fibers; however, these stress fibers were not seen following knockdown of *SUN1/2* and subsequent LIV. Previous work in fibroblasts demonstrated that the stiffness of the cell membrane was impaired following depolymerization of the actin cytoskeleton.^64^ In addition, results in mesenchymal stem cells found that vibration signals enhanced membrane stiffness by 24% compared to controls, which was associated with increases in signaling pathways leading to osteogenic responses.^13^ Taken together, our data suggest that application of mechanical stimuli increase cell membrane stiffness of breast cancer cells, possibly accounting for the decreased invasion and release of osteolytic factors. Furthermore, the LINC nuclear complex is necessary to mediate these beneficial effects of LIV, where loss of SUN1 and SUN2 prevent the ability of LIV to influence cell stiffness.

There were several limitations of the current study. First, the data are from in vitro assays, thus additional work in vivo will confirm the impact of LIV on breast cancer metastases and signaling to osteoclasts. Second, in some assays knockdown of *SUN1/2* resulted in increased responses in non-vibrated cells, such as in the invasion assay shown in Fig. 7a. These changes following knockdown of SUN1/2 may have influenced the data interpretation. While the primary focus of this work was to determine how the LINC complex regulates the responses of cancer cells to LIV, the increase in invasion following siSUN1/2 suggests that nucleo-cytoskeletal connectivity directly influences metastatic properties. The influence of the LINC complex on the metastatic potential of breast cancer cells has been suggested in other work.^20^ Lastly, the majority of assays were completed with ~4 biological replicates, representing a relatively small sample size. While our analyses demonstrate large significant differences for most assays, a larger sample size may have captured additional differences between samples.

These studies highlight the mechanisms through which mechanical force, a principal component of physical exercise, regulate the invasive potential of cancer cells and their ability to release paracrine signals that influence cancer progression. The role of the nucleus as a mechanosensory apparatus is just beginning to be appreciated, as is the function of the LINC nuclear complex in tumor pathogenicity. However, the use of exogenously applied mechanical forces may be an addition to the therapeutic armamentarium to both reduce tumor progression and prevent musculoskeletal compromise.

## Materials and methods

### Reagents

Fetal bovine serum (FBS) was obtained from Atlanta Biologicals (Atlanta, GA). Culture media, trypsin-EDTA, and antibiotics were purchased from Invitrogen (Carlsbad, CA). iTaq universal SYBR green qPCR mastermix (cat# 172–5121) was purchased from Bio-Rad (Hercules, CA). Recombinant human TGFβ-1 (cat# 240B002) was purchased from R&D systems (Minneapolis, MN). Alexa 488-conjugated phalloidin (cat# A12379) was purchased from Invitrogen.

### Cells and culture conditions

Human breast cancer cell lines, MDA-MB-231, MCF-7, and RAW 264.7 cells were purchased from the American Tissue Culture Collection (ATCC, Manassas, VA). MDA-MB-231 cells were cultured in Dulbecco’s Modified Essential Medium (DMEM) supplemented with FBS (10%, v/v) and penicillin/streptomycin (100 μg·mL^−1^). MCF-7 cells were cultured in mammary epithelial cell growth basal medium (MEBM) supplemented with the MEGM SingleQuots™ kit per manufacturer’s protocol. Murine RAW 264.7 macrophage cells were cultured in DMEM containing FBS (10%, v/v), penicillin/streptomycin (100 μg·mL^−1^), and recombinant RANKL (75 ng·mL^−1^). Cells were seeded to densities specific to each protocol.

### Real time PCR

Total RNA was isolated from cells using RNeasy kit (Qiagen, Germantown, MD), reverse transcribed, and genes were amplified with a BioRad CFX Connect™ qPCR machine, using gene-specific primers, as previously described.^65^ PCR products were normalized to *GAPDH* and quantified using the ΔΔCT method (denoted as ΔΔCq).

### siRNA-mediated knockdown

MDA-MB-231 cells were transfected with gene-specific siRNA or control siRNA (20 nmol·L^−1^) using PepMute Plus transfection reagent (SignaGen Labs, Rockville, MD). After 18 h of transfection, media was replaced with fresh DMEM containing FBS (10%, v/v) and penicillin/streptomycin (100 μg·mL^−1^). LIV treatment was initiated 1–2 h after media was replaced. The following Stealth Select siRNAs (Invitrogen) were used in this study: negative control for *SUN1:* 5′-AAGGTTGCGTGGTTATAAACGCCTG-3′; *SUN1:* 5′-CAGGACGTGTTTAAACCCACGACTT-3′; negative control for *SUN2:* 5′-GCATTACCACCGTCCTTTCGAGGTT-3′; *SUN2:* 5′-GCAGACATTCCACCCTGCTTTGGTT-3′.

### Antibodies

Antibodies targeting SUN1 (cat# ab12) and SUN2 (cat# ab124916) were purchased from Abcam (Cambridge, MA). The Nesprin1 Ab (cat# MA5-18077) was from Invitrogen. The anti-β actin Ab (cat# 5125) was purchased from Cell Signaling (Danvers, MA).

### Low magnitude mechanical force

Low magnitude mechanical forces were applied in the form of LIV using a custom-designed platform, as previously described.^25^ Individual culture dishes were placed on the vertically oscillating platform at room temperature (RT). Cells were stimulated at a frequency of 90 Hz at a magnitude of 0.300 g ± 0.025 g, where 1 g is equal to the earth’s gravitational field or 9.8 m·s^−2^. LIV was applied in 20-min bouts, once- or twice-daily for 3 days. Twice-daily bouts were separated by 3 h of rest. Non-vibrated control cells were placed on the vibration platform that was not turned on.

### Cell viability and apoptosis assays

Cell viability was assessed using the CellTiter96® Aqueous One Solution Assay (cat# G3582), which utilizes 3-(4,5-Dimethylthiazol-2-yl)-2,5-Diphenyltetrazolium Bromide (MTT) (Promega, Madison WI). The assay was carried out according to the manufacturer’s protocol. Colorimetric changes were measured at 490 nm using a spectrophotometer. Apoptosis was quantified using the BD Pharmingen™ (San Jose, CA) PE Annexin V detection kit (cat# 559763). Cells were stained according to the manufacturer’s protocol and Annexin V labeled cells were quantified using a BD Accuri C6 flow cytometry machine.

### Transwell assays

Invasion of MDA-MB-231 cells was quantified using Transwell^®^ membrane inserts with a pore size of 8.0 μm (Corning^®^, Corning, NY). Cells first were seeded to 6-well dishes, exposed to LIV or non-LIV conditions for the appropriate number of days. At least 2 h prior to seeding of cells on to transwell inserts, Matrigel^®^ (Corning^®^, cat# 356234) was diluted with DMEM (1:5) and added (100 μL) to the upper chamber of insert within the 24-well dish. Following LIV, cells were trypsinized and seeded (50 000 cells per well) onto Matrigel^®^-containing upper chamber of the transwell inserts. Cells were suspended in DMEM containing FBS (1%, v/v). The lower chamber contained DMEM with FBS (10%, v/v), P/S (1%, v/v) and collagen (rat tail, type I, cat# 354236, 40 ng·mL^−1^). Cells within the transwell insert were incubated at 37 °C for 24 h, then fixed with methanol (100%, v/v) for 10 min and stained with crystal violet (0.5%, w/v) for 10 min. Cell number and area were quantified under light microscopy.

### Western blotting

Whole cell lysates were prepared using radio immunoprecipitation assay (RIPA) lysis buffer (150 mmol·L^−1^ NaCl, 50 mmol·L^−1^ Tris HCl, 1 mmol·L^−1^ EGTA, 0.24% sodium deoxycholate,1% Igepal, pH 7.5) containing NaF (25 mmol·L^−1^) and Na_3_VO_4_ (2 mmol·L^−1^). Aprotinin, leupeptin, pepstatin, and phenylmethylsulfonylfluoride (PMSF) were added fresh, just prior to lysis. Whole cell lysates (20 μg) were separated on polyacrylamide gels (4%–12%) and transferred to polyvinylidene difluoride (PVDF) membranes. Membranes were blocked with milk (5%, w/v) diluted in TBS-T. Blots then were incubated overnight at 4 ˚C with the appropriate primary antibodies. Blots were washed and incubated with horseradish peroxidase-conjugated secondary antibody (1:5 000 dilution) (Cell Signaling) at RT for 1 h. ECL plus was used to detect chemiluminescence (Amersham Biosciences, Piscataway, NJ). Images were developed and acquired with an iBright CL1000 machine (Applied Biosystems), and densitometry was determined using ImageJ software version 1.45s (NIH).

### Immunofluorescence

Following LIV and/or siRNA transfection, cells were fixed with paraformaldehyde (4%, v/v) for 20 min, permeabilized with Triton X-100 (0.1%, v/v) for 5 min at RT, and donkey serum (5%, v/v) blocking buffer diluted in TBS-T was added for 30 min, as previously described.^66^ Cells were incubated with phalloidin-conjugated Alexa Fluor-488 (Invitrogen) diluted in TBS (1:100) 30 min at RT. Cells were washed, covered, and sealed with mounting medium containing DAPI (Invitrogen).

### Osteoclast differentiation and pit assays

Osteoclast differentiation was determined using murine RAW 264.7 macrophage cells, which were seeded onto 6-well dishes (50 000 cells per well). Cells were maintained in DMEM containing RANKL (75 ng·mL^−1^), which was replaced with CM from MDA-MB-231 cells after 24 h. A 50% mixture of CM and DMEM was added from each group (+/−LIV and +/−si*SUN1/2*) with RANKL (75 ng·mL^−1^). Following 4 days of differentiation, cells were stained with TRAP (TRAP, Sigma, Cat# 386A-1KT) according to the manufacturer’s protocol. Cells were considered to be osteoclasts if they stained for TRAP and had three or more nuclei.

Resorption activity of osteoclasts, in the presence of CM from MDA-MB-231 cells, was quantified using the Osteo Assay (Corning, Corning NY) containing hydroxyapatite. In brief, 5 000 RAW 264.7 cells were seeded onto the 96-well Osteo Assay plate containing 200 μL of DMEM with RANKL (75 ng·mL^−1^). The next day, half the volume of media was replaced with CM from MDA-MB-231 cells for each condition. Media was replaced every two days. At day 4, media was removed and 100 μL of bleach (10%, v/v) was added for 5 min at RT. Bleach was removed and cells were washed twice with water. Formation of pits in the hydroxyapatite were visualized under light microscopy and quantified with ImageJ.

### Determination of elastic modulus using atomic force microscopy

Cells were indented in media at RT using a BioScope Catalyst AFM (Bruker, Santa Barbara, CA). The AFM was mounted on a Leica DMI3000 inverted microscope (Leica Biosystems Inc., Buffalo Grove, IL), facilitating accurate placement of the AFM probe over individual cells. Indentations were carried out using a spherical borosilicate bead (5 μm diameter) mounted on a gold-coated silicon nitride cantilever (Novascan Technologies, Inc., Boone, IA). Prior to indenting, probes were pushed onto a glass surface and the deflection of the cantilever was used to measure the cantilever’s deflection sensitivity (nm/V). The cantilever’s spring constant (~0.07 N·m^−1^) was then determined using the thermal tuning method. The light microscope was used to navigate to randomly selected cells. The apex of the cantilever (where the bead is attached) was placed directly above the nucleus, then the AFM was engaged. A single ramp was performed using a trigger force of 1 nN at a speed of 0.5 Hz.

Analysis was performed using Nanoscope Analysis v1.70 (Bruker). A linear baseline correction was fit from 30% to 80% of the retraction curve. Since Poisson’s ratio is not fully understood for cells, reduced elastic modulus (*E*_r_) was fit for each unloading curve in a region spanning from 20% to 75% of the maximum force using the Hertz model of contact between a rigid sphere and an elastic half space:

1 $$F = \frac{4}{3} \cdot E_r \cdot \sqrt r \cdot \delta ^{\frac{3}{2}}.$$

In Eq. 1, *F* is force exerted on the cell, *r* is the radius of curvature of the probe and δ is sample deformation. Only indents with goodness of fits (i.e., *r*^2^) >0.97 were included for analysis.

### Statistical analysis

Statistical variance was expressed as the means ± standard error of the mean (SEM). Evaluation of statistical significance performed by one-way analysis of variance (ANOVA), two-way ANOVA, or student’s *t*-test, as appropriate (Prism GraphPad, La Jolla, CA). For AFM assays, two-way ANOVA was used followed by two-stage linear step-up procedure of Benjamini, Krieger, and Yekutieli to control for false discovery rate. Values were considered significant if *P* ≤ 0.05. All experiments were replicated at least three times using unique biological replicates to assure reproducibility. Biological replicates were generated by newly passaging and seeding cells or using a fresh aliquot of cells for each assay. Densitometry data from Western blots were compiled from four biological replicates.

## Supplementary information

Suppl Figures

Suppl Figure Legends
