# Supplementary figures and images for: Mechanical suppression of breast cancer cell invasion and paracrine signaling to osteoclasts requires nucleo-cytoskeletal connectivity
Source: Bone Res. 2020 Nov 17;8:40. doi: 10.1038/s41413-020-00111-3 (PMC7673025; doi:10.1038/s41413-020-00111-3)

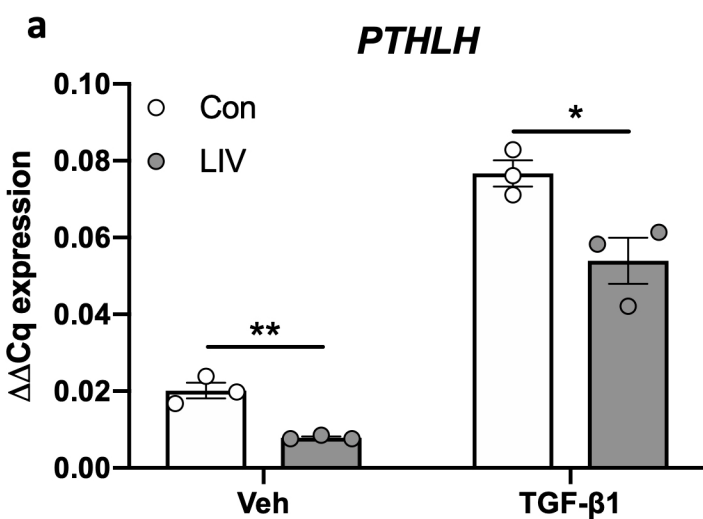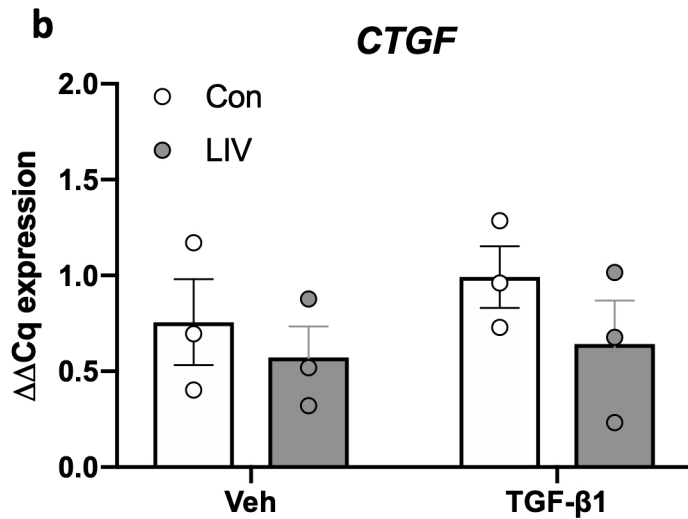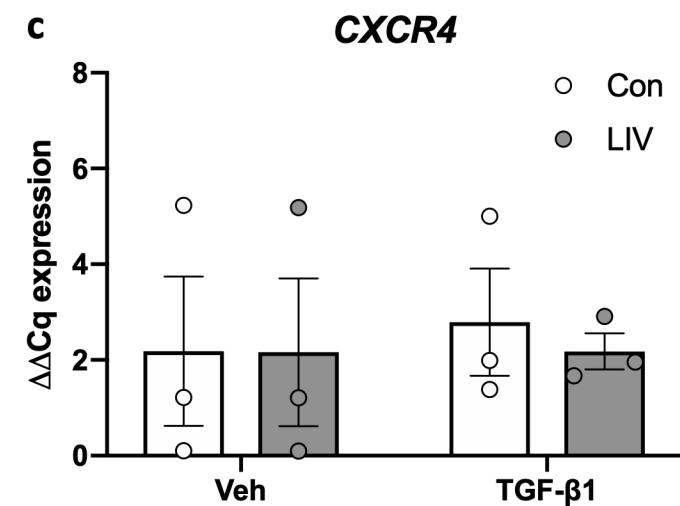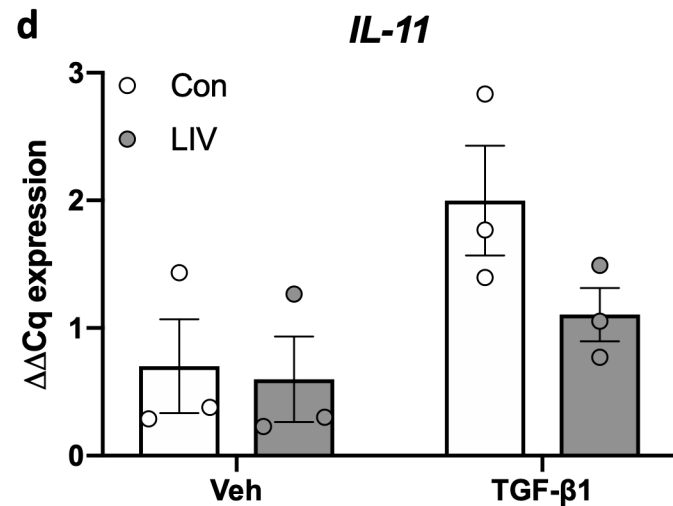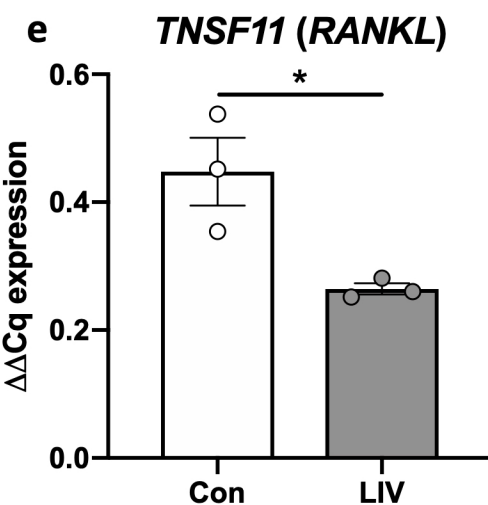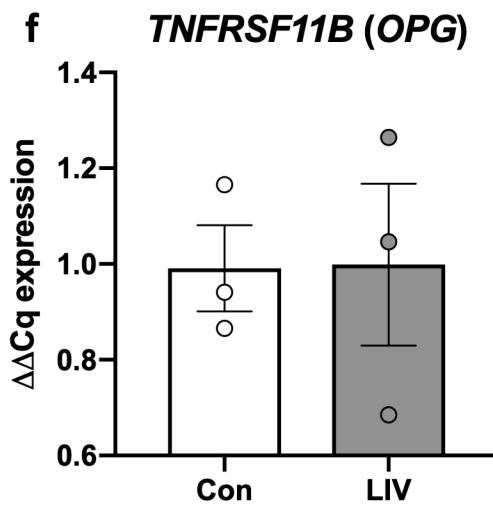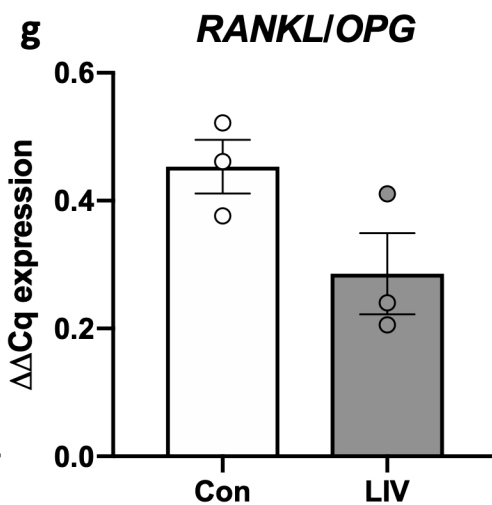

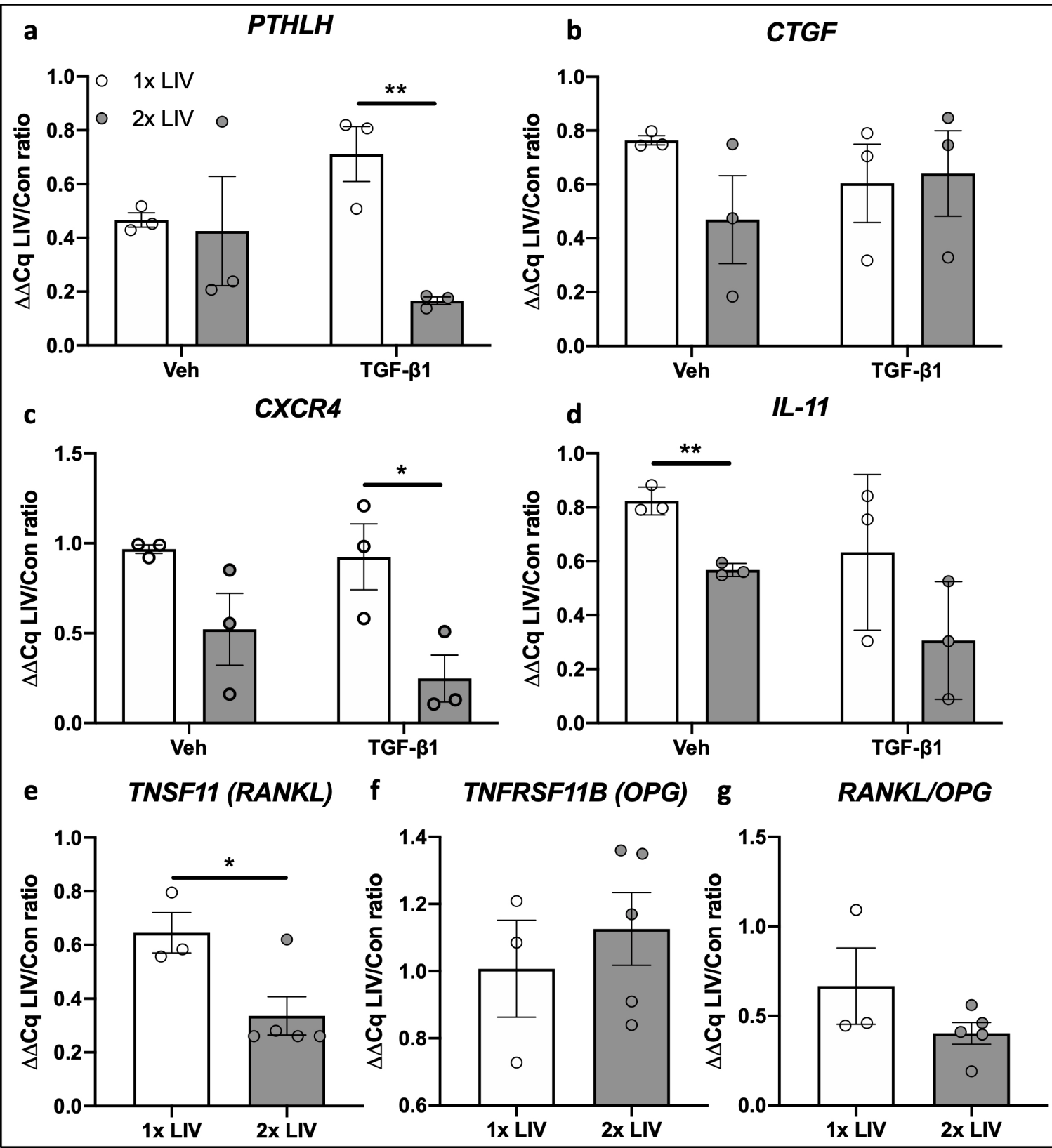

SUN1

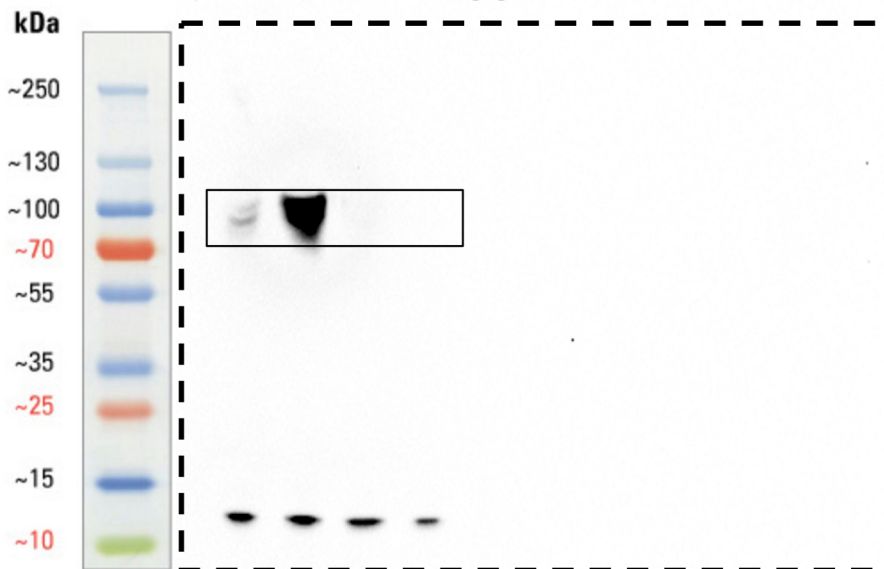

SUN2

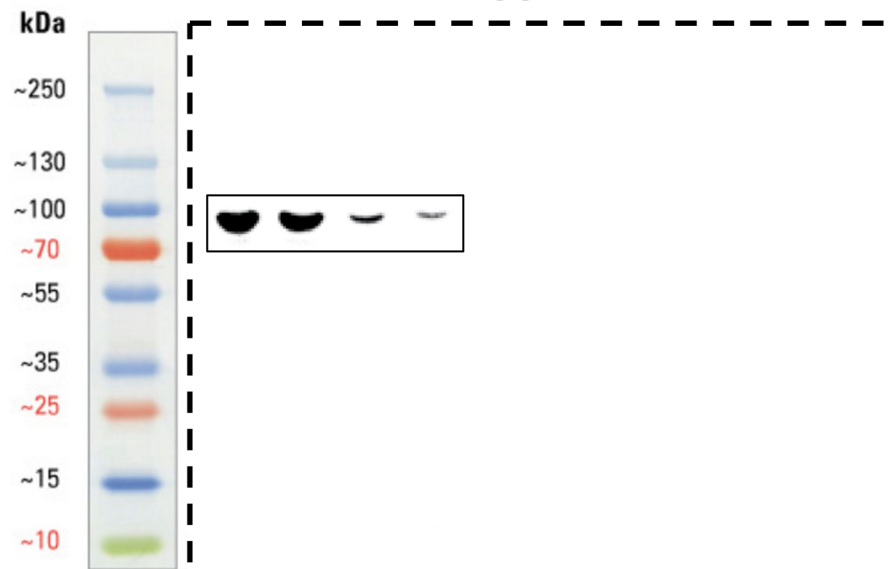 $\beta$ -actin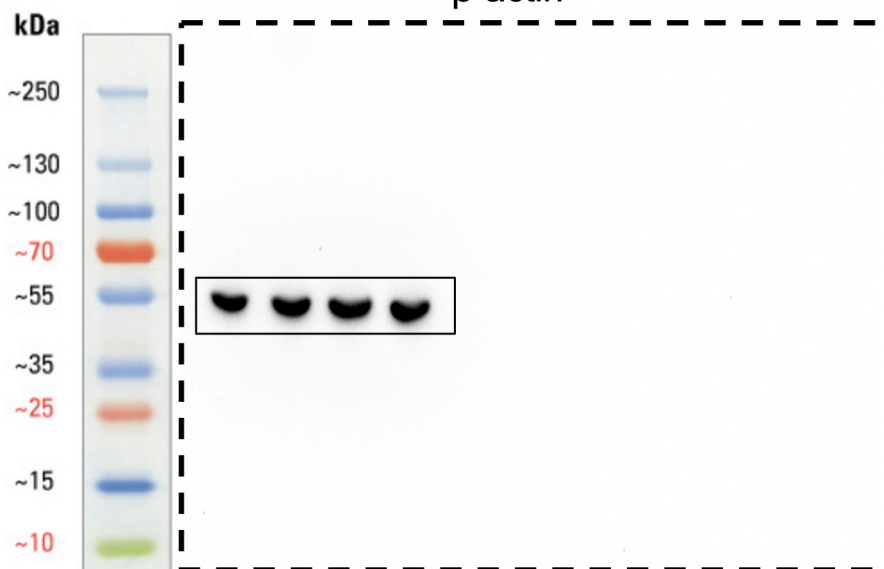 $\beta$ -actin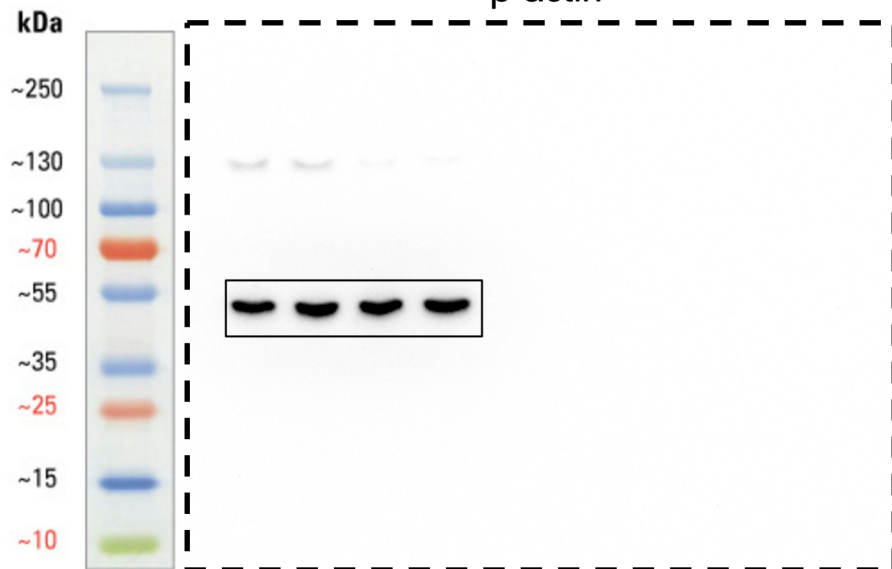

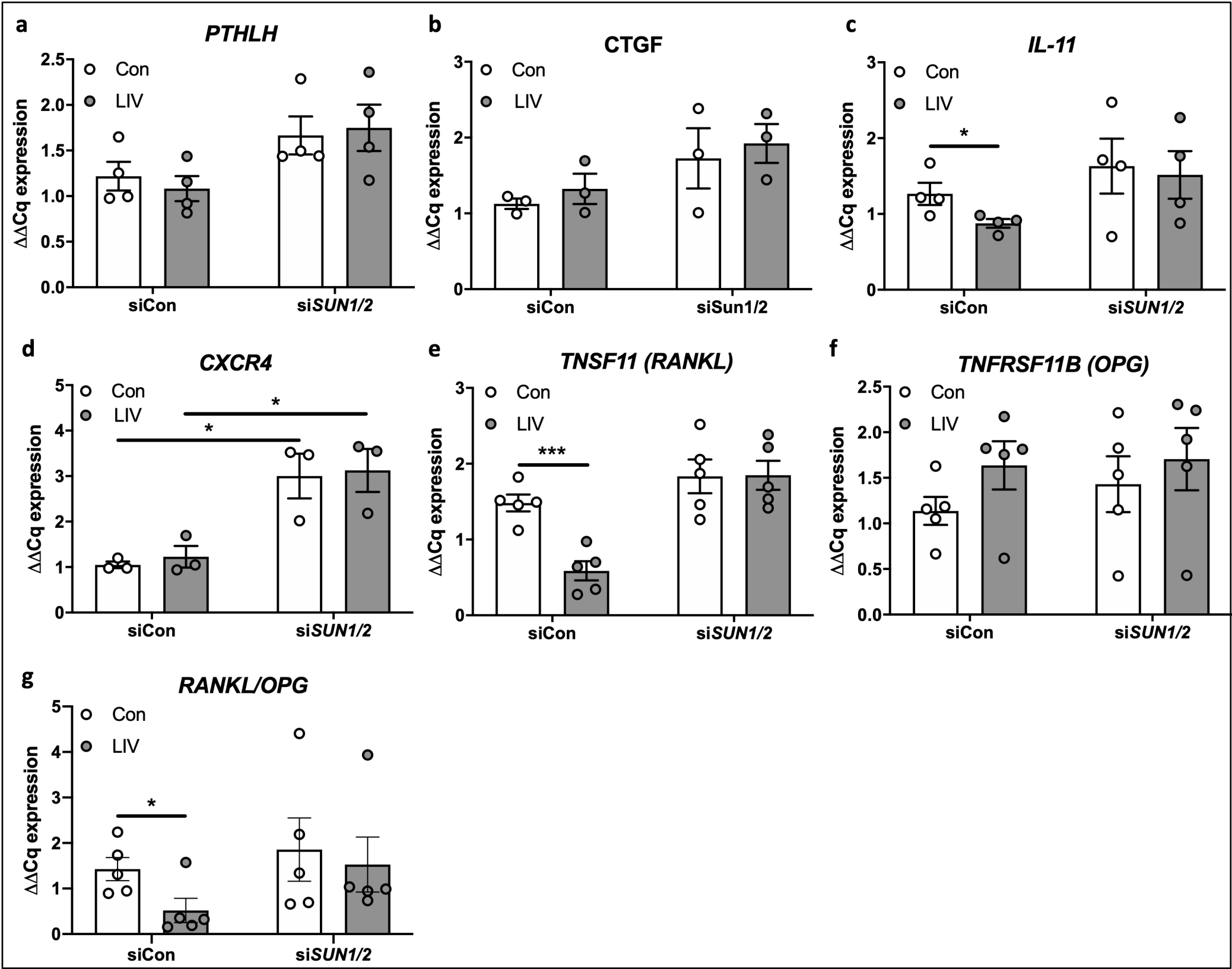

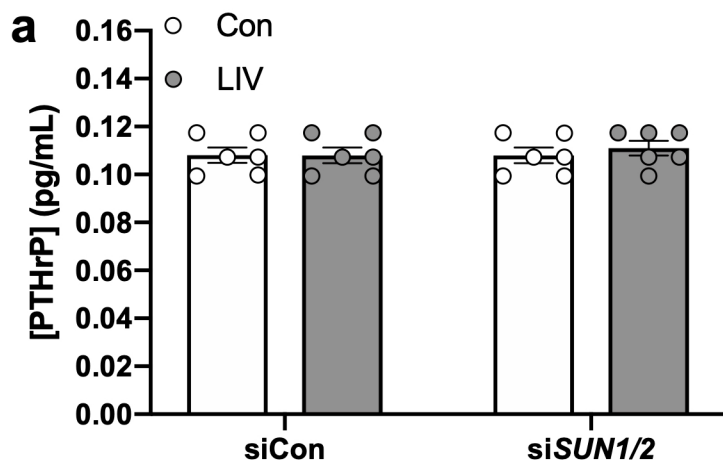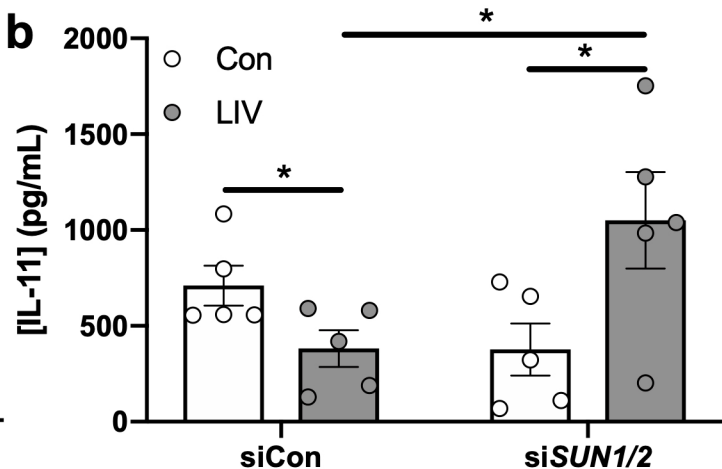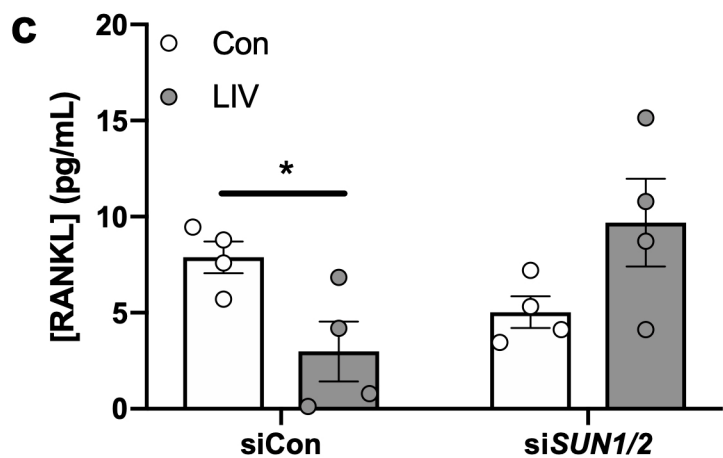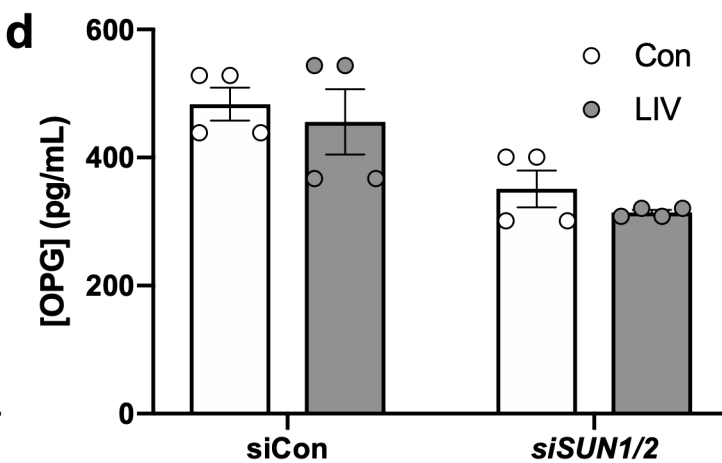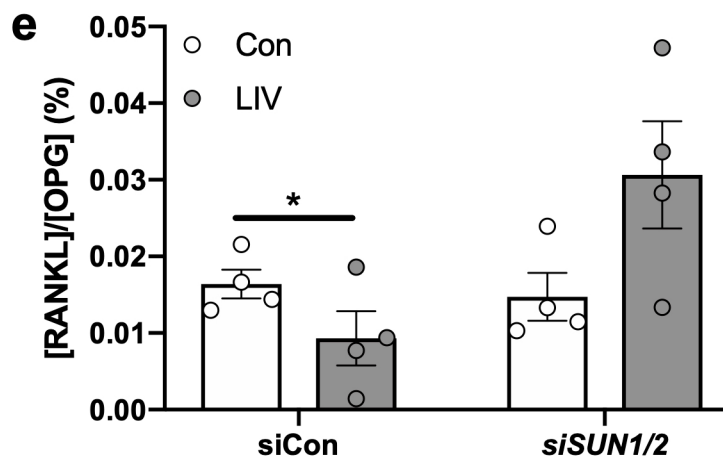

**Con**

**LIV**

**siCon**

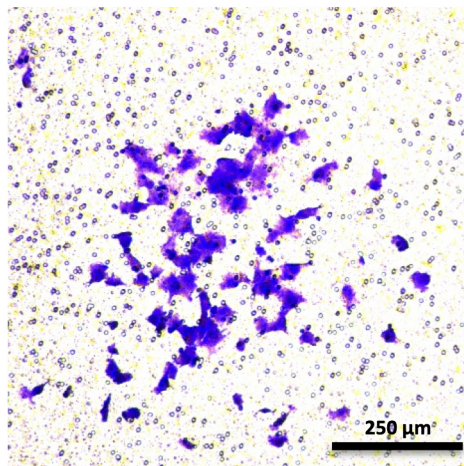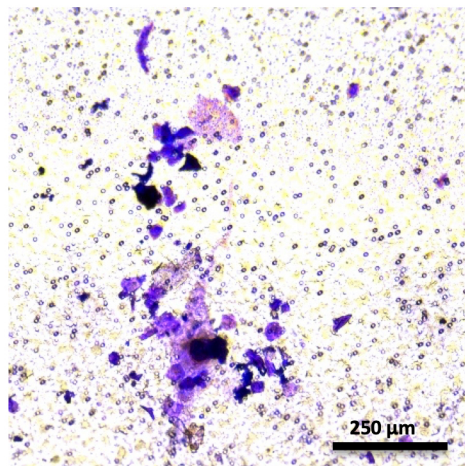

**siSUN1/2**

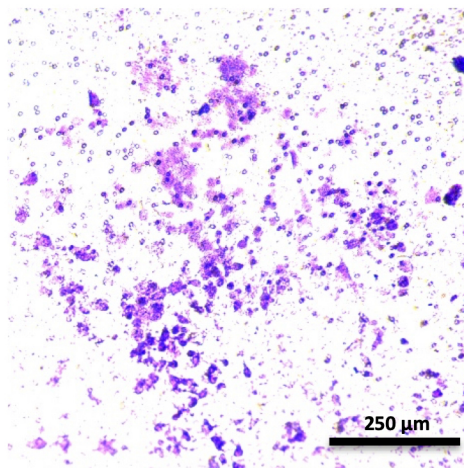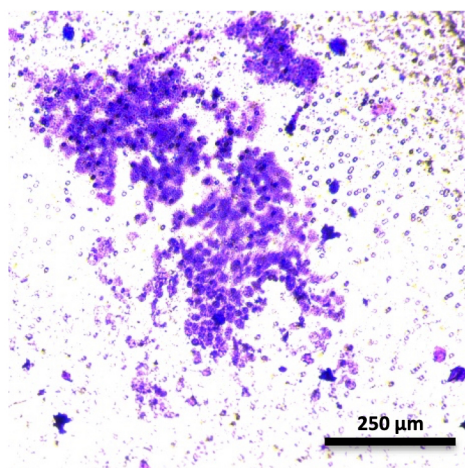

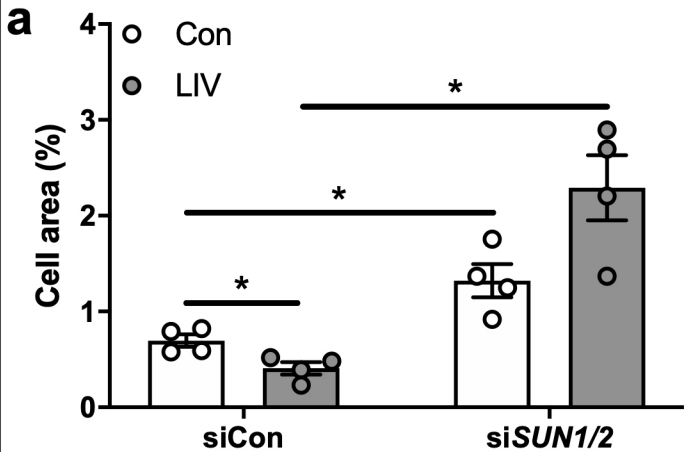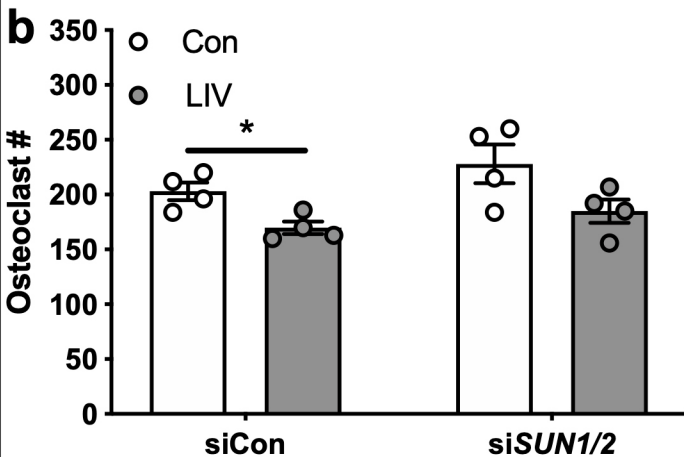

**Con**

**LIV**

**siCon**

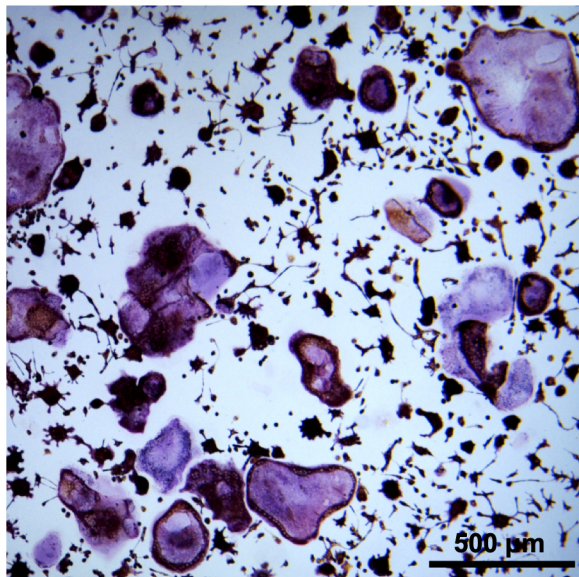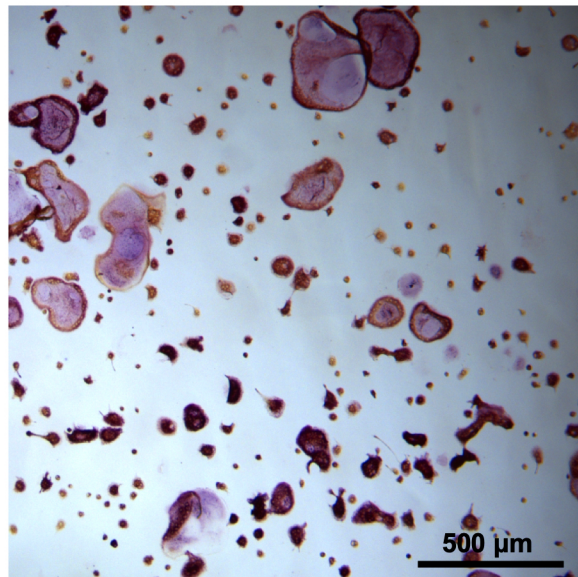

**siSUN1/2**

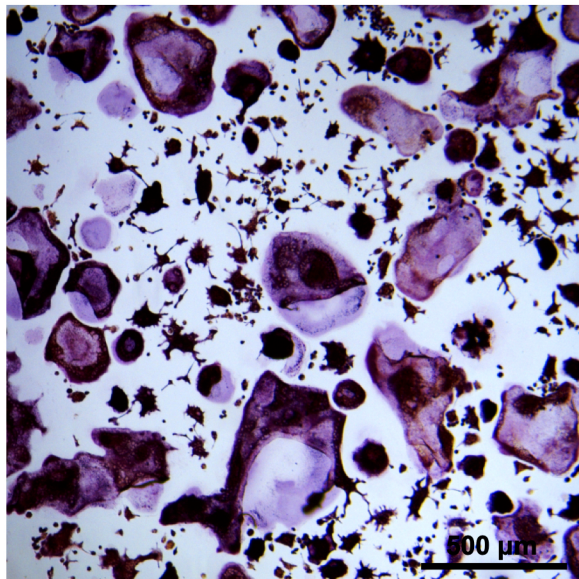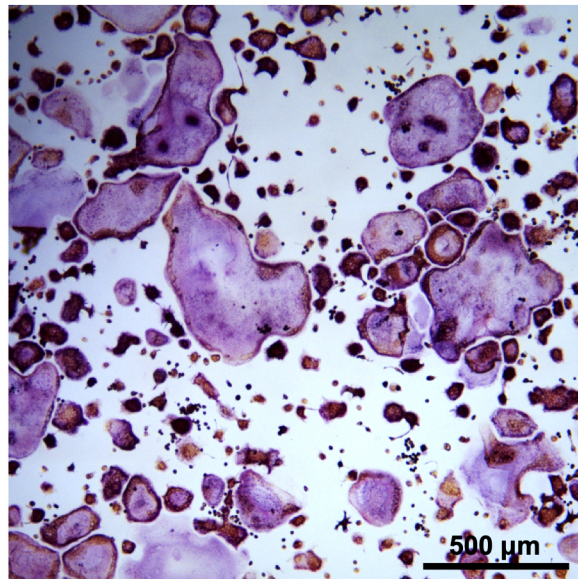

**Con**

**LIV**

**siCon**

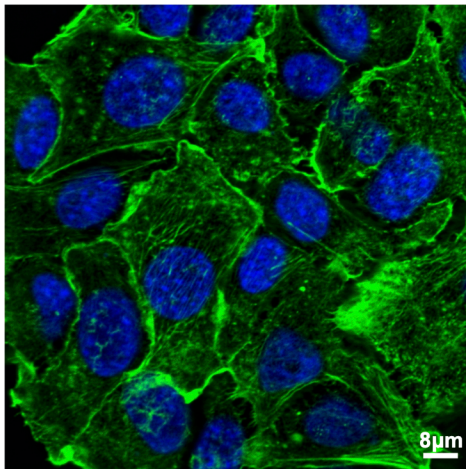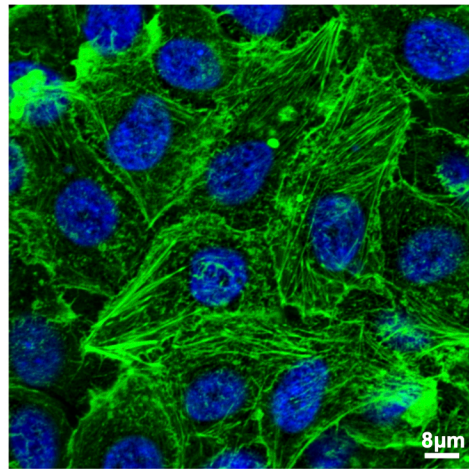

**siSUN1/2**

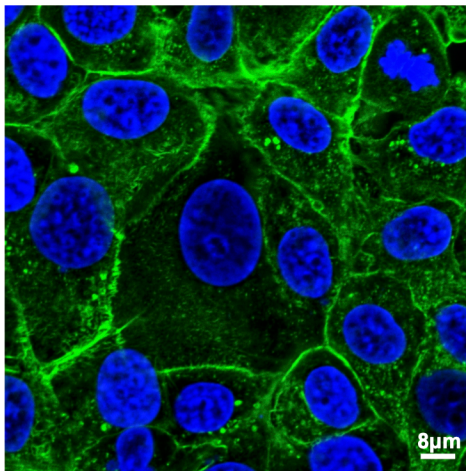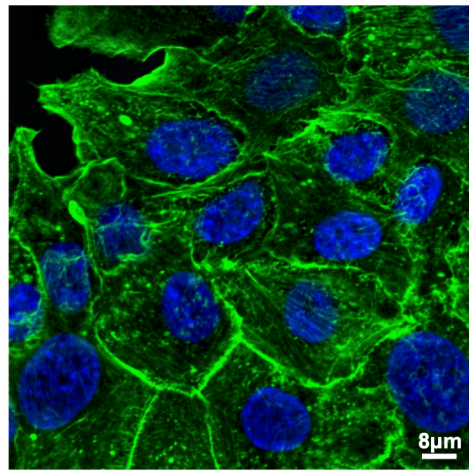

Supplement: Supplementary file 1 — Suppl Figures [file 41413_2020_111_MOESM1_ESM.pdf]
