# Supplementary material for: Mechanical suppression of breast cancer cell invasion and paracrine signaling to osteoclasts requires nucleo-cytoskeletal connectivity
Source: Bone Res. 2020 Nov 17;8:40. doi: 10.1038/s41413-020-00111-3 (PMC7673025; doi:10.1038/s41413-020-00111-3)
Supplement: Supplementary file 2 — Suppl Figure Legends [file 41413_2020_111_MOESM2_ESM.docx]

**Figure S1** Expression of osteolytic factors following once-daily LIV. MDA-MB-231 cells were treated with PBS (Veh) or TGF-β1 and exposed to non-vibration control conditions (Con) or LIV once-daily for 3 days. qPCR analyses were normalized to *GAPDH*. Genes surveyed included (a) parathyroid hormone-related protein (*PTHLH*) (n=3), (b) connective tissue growth factor (*CTGF*) (n=3), (c) C-X-C chemokine receptor type 4 (*CXCR4*) (n=3), (d) interleukin 11 (*IL-11*) (n=3), (e) receptor activator of nuclear factor kappa-B ligand (TNFSF11, *RANKL*) (n=3), and (f) osteoprotegerin (*TNFRSF11B, OPG*) (n=3). (g) Quantification of the ratio of *RANKL* to *OPG* mRNA (n=3). n=3 for all assays. Multiple t-test or student’s t-test p values: *p<0.05, **p<0.01.

**Figure S2** Expression of osteolytic factors by qPCR comparing once- and twice-daily LIV treatments. MDA-MB-231 cells were treated with PBS (Veh) or TGF-β1 and exposed to non-vibration control conditions (Con) or LIV once-daily for 3 days. *GAPDH* was used as a housekeeping gene. qPCR values were normalized to the non-vibrated control reference. Genes surveyed included (a) parathyroid hormone-related protein (*PTHLH*), (b) connective tissue growth factor (*CTGF*), (c) C-X-C chemokine receptor type 4 (*CXCR4*), (d) interleukin 11 (*IL-11*), (e) receptor activator of nuclear factor kappa-B ligand (TNFSF11, *RANKL*), and (f) osteoprotegerin (*TNFRSF11B, OPG*). (g) Quantification of the ratio of *RANKL* to *OPG* mRNA. n=3-5 for each condition. Multiple t-test or student’s t-test p values: *p<0.05, **p<0.01.

**Figure S3** Original un-cropped images of Western blots shown in figures 6f and 6g. Lysates were collected using RIPA buffer following siRNA knockdown of *SUN1* or *SUN2* (or using control siRNAs) or exposure to twice-daily LIV (or non-LIV conditions), as shown in figure 6. Dotted lines denote the full outline of the entire PVDF membrane.

**Figure S4** qPCR analysis of MCF-7 cells. MCF-7 cells were transfected with siRNAs targeting *SUN1/2* and treated with LIV or control conditions twice a day for 3 days. Expression was normalized to *GAPDH*. Genes surveyed included (a) parathyroid hormone-related protein (*PTHLH) (n=4)*, (b) connective tissue growth factor (*CTGF) (n=3)*, (c) interleukin 11 (*IL-11*) *(n=4)*, (d) C-X-C chemokine receptor type 4 (*CXCR4*) *(n=3)*, (e) receptor activator of nuclear factor kappa-B ligand (TNFSF11, *RANKL*) *(n=5)*, and (f) osteoprotegerin (*TNFRSF11B, OPG*) *(n=5)*. (g) Quantification of the ratio of *RANKL* to *OPG* mRNA. n=3-5 for all assays. Multiple t-test or student’s t-test p values: *p<0.05, ***p<0.001.

**Figure S5** Secretion of osteolytic factors into conditioned media. Conditioned media was collected from MDA-MB-231 cells following transfection with control siRNAs (siCon) or siRNAs targeting *SUN1* and *SUN2* (siSUN1/2) and non-vibration control conditions (Con) or twice-daily LIV for 3 days (LIV). ELISA assays were performed for the following proteins (a) PTHrP (n=6), (b) IL-11 (n=5), (c) RANKL (n=4), and (d) OPG (n=4). (e) The ratio of RANKL to OPG protein was determined. Multiple t-test or student p values: *p<0.05.

**Figure S6** Mechanical suppression of MDA-MB-231 invasion requires *SUN1* and *SUN2*. Representative images showing crystal violet staining (purple) of MDA-MB-231 cells that have invaded through Matrigel^®^ and penetrated through the trans-well membrane. Cells were exposed to twice-daily LIV for 3 days (LIV) after transfection with control siRNA oligos or siRNAs targeting *SUN1* and *SUN2*. Images are representative of four biological replicates. Quantification of cell invasion provided in figure 7a.

**Figure S7** LIV suppresses invasion and production of osteolytic factors from MCF-7 cells. (a) Quantification of cell area invading through trans-well membrane under control (Con, no LIV) or twice-daily LIV (LIV) conditions. MCF-7 cells were transfected with control siRNA oligos (siCon) or with siRNAs targeting both *SUN1* and *SUN2* (si*SUN1/2*). (b) Quantification of the number of osteoclasts following exposure of RAW 264.7 cells to conditioned media from MCF-7 cells that received LIV twice-daily for 3 days and transfected with control siRNA sequences (siCon) or siRNAs targeting *SUN1* and *SUN2* (si*SUN1/2*) prior to LIV. Data compiled from 4 biological replicates and measured using ImageJ. Two-way ANOVA p values: *p<0.05.

**Figure S8** The LINC complex regulates the ability of mechanical force to alter secretion of factors from breast cancer cells that influence osteoclast formation. Representative images showing RAW 264.7 cells stained with TRAP following the addition of conditioned media from MDA-MB-231 cells that were transfected with control siRNAs or siRNAs targeting *SUN1* and *SUN2* and exposed to non-vibration control conditions (Con) or twice-daily LIV for 3 days (LIV). A 50:50 mixture of conditioned media and growth media (DMEM) was added to each well of RAW 264.7 cells for 4 days prior to TRAP staining. Quantification of osteoclast number, following TRAP staining, is shown in figure 7b.

**Figure S9** LIV-induced actin stress fiber formation in MCF-7 cells. Representative images of MCF-7 cells exposed to non-vibration control conditions (Con), twice-daily LIV, and either control siRNAs or siRNA oligos targeting *SUN1* and *SUN2* (si*SUN1/2*). For each condition, cells were fixed following a 3-hour rest period after the last LIV bout and incubated with Phalloidin-conjugated Alexa-Fluor 488 and Dapi to stain for filamentous actin (green) and nuclei (blue) respectively. Images are representative of three biological replicates.
